# Contribution of rare homozygous and compound heterozygous *VPS13C* missense mutations to dementia with Lewy bodies and Parkinson’s disease

**DOI:** 10.1186/s40478-021-01121-w

**Published:** 2021-02-12

**Authors:** Stefanie Smolders, Stéphanie Philtjens, David Crosiers, Anne Sieben, Elisabeth Hens, Bavo Heeman, Sara Van Mossevelde, Philippe Pals, Bob Asselbergh, Roberto Dos Santos Dias, Yannick Vermeiren, Rik Vandenberghe, Sebastiaan Engelborghs, Peter Paul De Deyn, Jean-Jacques Martin, Patrick Cras, Wim Annaert, Christine Van Broeckhoven, BELNEU consortium, Chris van der Linden, Emke Maréchal, Rik Vandenberghe, Bruno Bergmans

**Affiliations:** 1 Center for Molecular Neurology, VIB, Antwerp, Belgium; 2 Institute Born-Bunge, Antwerp, Belgium; 3 University of Antwerp, Antwerp, Belgium; 4 Department of Neurology, University Hospital Antwerp, Edegem, Belgium; 5 Department of Neurology, Hospital Network Antwerp, Antwerp, Belgium; 6 Department of Neurology, University Hospital Ghent and University of Ghent, Ghent, Belgium; 7 Department of Neurosciences, Center for Brain and Disease Research VIB, KU Leuven, Leuven, Belgium; 8 Department of Neurology, University Hospitals Leuven, Leuven, Belgium; 9 Department of Neurosciences, KU Leuven, Leuven, Belgium; 10 Department of Neurology, UZ Brussel and Center for Neurosciences, Vrije Universiteit Brussel (VUB), Brussels, Belgium; 11 Department of Biomedical Sciences, Neurodegenerative Brain Diseases, University of Antwerp, Antwerp, Belgium

**Keywords:** Lewy body disease, Dementia with lewy bodies, DLB, Parkinson’s disease, PD, Vacuolar protein sorting 13 homolog C, VPS13C, Recessive inheritance, Missense mutations, Loss-of-function

## Abstract

Dementia with Lewy bodies (DLB) and Parkinson’s disease (PD) are clinically, pathologically and etiologically disorders embedded in the Lewy body disease (LBD) continuum, characterized by neuronal α-synuclein pathology. Rare homozygous and compound heterozygous premature termination codon (PTC) mutations in the Vacuolar Protein Sorting 13 homolog C gene (*VPS13C*) are associated with early-onset recessive PD. We observed in two siblings with early-onset age (< 45) and autopsy confirmed DLB, compound heterozygous missense mutations in *VPS13C*, p.Trp395Cys and p.Ala444Pro, inherited from their healthy parents in a recessive manner. In lymphoblast cells of the index patient, the missense mutations reduced VPS13C expression by 90% (*p* = 0.0002). Subsequent, we performed targeted resequencing of *VPS13C* in 844 LBD patients and 664 control persons. Using the optimized sequence kernel association test, we obtained a significant association (*p* = 0.0233) of rare *VPS13C* genetic variants (minor allele frequency ≤ 1%) with LBD. Among the LBD patients, we identified one patient with homozygous missense mutations and three with compound heterozygous missense mutations in *trans* position, indicative for recessive inheritance. In four patients with compound heterozygous mutations, we were unable to determine *trans* position. The frequency of LBD patient carriers of proven recessive compound heterozygous missense mutations is 0.59% (5/844). In autopsy brain tissue of two unrelated LBD patients, the recessive compound heterozygous missense mutations reduced VPS13C expression. Overexpressing of wild type or mutant *VPS13C* in HeLa or SH-SY5Y cells, demonstrated that the mutations p.Trp395Cys or p.Ala444Pro, abolish the endosomal/lysosomal localization of VPS13C. Overall, our data indicate that rare missense mutations in *VPS13C* are associated with LBD and recessive compound heterozygous missense mutations might have variable effects on the expression and functioning of VPS13C. We conclude that comparable to the recessive inherited PTC mutations in *VPS13C,* combinations of rare recessive compound heterozygous missense mutations reduce VPS13C expression and contribute to increased risk of LBD.

## Introduction

Lewy body diseases (LBD) are a heterogeneous group of neurodegenerative brain diseases characterized by the presence of Lewy bodies and Lewy neurites, mainly composed of aggregated α-synuclein in neurons. Two disorders with substantial clinical, pathological and etiological overlap are at the two extremes of the LBD continuum: dementia with Lewy bodies (DLB) and Parkinson’s disease (PD) [14, 43]. In DLB patients, the early cognitive deterioration often resembles other dementias, but the presence of parkinsonism, visual hallucinations and delusions, and rapid eye movement (REM)-sleep behavior disorder distinguishes DLB [28]. PD is primarily characterized by manifestation of parkinsonism, however, mild cognitive impairment and subsequent dementia are observed in roughly 30% of PD patients [1]. Lewy body inclusions in PD brains are restricted to the brainstem and the limbic system, whereas the Lewy body pathology extends to the neocortex in DLB brains [8, 40]. DLB and PD have prevalences of 0.37% and 1–2%, in people aged 65 years and older [35, 41]. Of all LBD patients, 85–95% are sporadic patients, nonetheless, families segregating LBD in a Mendelian manner have been described [3, 5, 6, 29]. Family-based and genome-wide association studies identified over 70 PD loci with variable genetic contributions to PD risk [3, 31]. A causal gene for DLB has not been identified yet. Targeted and genome-wide association studies associated the *SNCA*, *GBA* and *APOE* loci with increased risk of developing DLB [9, 18]. Acquired knowledge of the proteins derived from causal or risk genes provided valuable insights into the underlying disease mechanisms of LBD [2, 4, 16, 34, 38, 44]. Nonetheless, causal genes in families and risk genes in patient cohorts are together only a minor fraction of the genetic etiology of LBD. In this study, we started from a family of healthy parents and two siblings with pathologically confirmed DLB at early-onset age (< 45 years). The affected siblings tested negative for mutations in known LBD genes. Whole genome sequencing (WGS) of the siblings revealed compound heterozygous coding variants in *VPS13C*, p.Trp395Cys and p.Ala444Pro missense mutations, which were inherited from their parents in a recessive manner. Homozyous and compound heterozygous loss-of-function (LOF) mutations due to premature termination codons (PTCs) were reported in recessive early-onset PD [11, 24, 37]. Since we showed that recessive compound heterozygous missense mutations in the siblings lead to 90% loss of VPS13C, we aimed at finding other homozygous or recessive compound heterozygous carriers in DLB and PD patient cohorts.

## Materials and methods

Note: Detailed protocols are available in the Additional file 1.

### Belgian patient and control cohorts

Members of the Belgian BELNEU consortium were involved in the recruitment of LBD patients at neurological centers associated with university or general hospitals in Belgium [26]. The LBD cohort comprised 844 LBD patients with a mean age at onset age (AAO) of 62.9 ± 11.8 years (Additional file 1: Table S1). In this cohort, 233 patients had a diagnosis of DLB, and 611 a diagnosis of PD. All patients underwent clinical examinations by a neurologist and neuroimaging. Detailed information on medical history of patient and family members were collected. A positive family history of disease was given if at least one first-degree relative was affected with a neurodegenerative brain disease. DLB patients were diagnosed in accordance with the established criteria for possible, probable or pathological DLB [27, 28], and PD patients according to the NINDS diagnostic criteria for PD [15]. A geographically matched control cohort consisted of 664 individuals with a mean age at inclusion (AAI) of 72.0 ± 9.4 years (Additional file 1: Table S1). Control individuals were recruited among healthy partners of patients visiting a memory clinic, and negative for neurological or psychiatric antecedents or neurological complaints, or community-recruited individuals scoring > 25 on a Montreal Cognitive Assessment (MoCA) [33] with a negative individual or familial history of neurodegenerative or psychiatric diseases.

### Whole genome sequencing and targeted resequencing

**Fig. 1:**
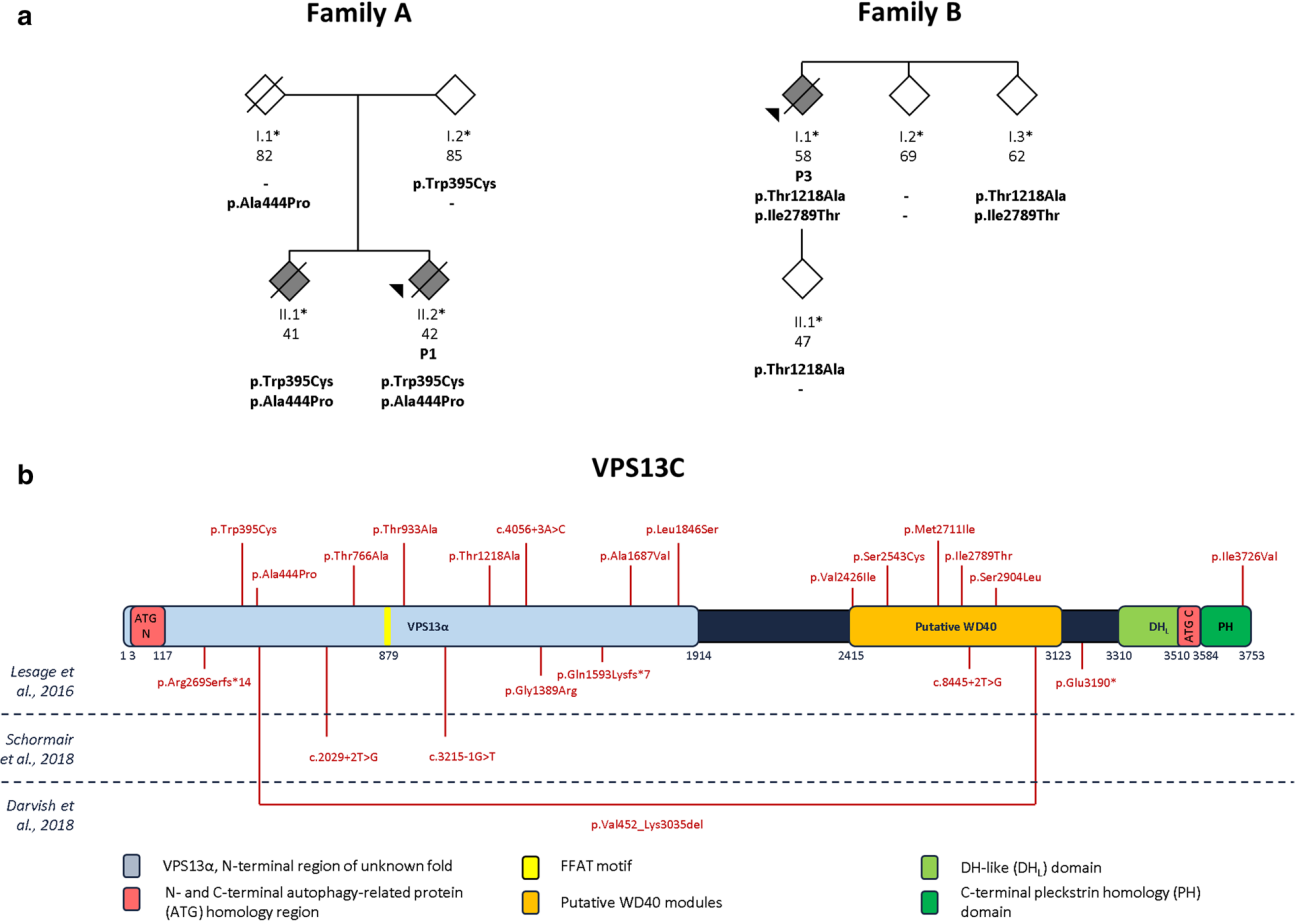
Homozygous and compound heterozygous *VPS13C* mutations in 2 Belgian LBD families and in a LBD cohort. **a** Pedigree of family A and B. Family A with two siblings affected with DLB and carriers of *trans* compound heterozygous rare mutations, p.Trp395Cys/p.Ala444Pro. The index patient P1 (black arrow), received a pathological diagnosis of diffuse LBD of the neocortical type. Family B with one affected patient (P3) with compound heterozygous mutations, p.Thr1218Ala/p.Ile2789Thr. Patient P3 had a pathological diagnosis of LBD, of the predominant amygdala type. Additionally, patient P3 had full AD pathology in all three neuropathological changes, A3B3C3 [20, 28]. To make the pedigrees anonymous, we used diamonds for the family members and the patients (black symbol) and we added to the pedigree only family members needed to show the cis/*trans* location of the *VPS13C* mutations. Slashed symbols indicate deceased family members. **b** Linear presentation of the VPS13C protein with domains based on [22], protein nomenclature according to NP_065872.1. Above, we positioned the homozygous or compound heterozygous mutations with a MAF of ≤ 1%, observed in the families and in the DLB and PD cohorts. The VPS13Cα domain is involved in transport of glycerophospholipids, the putative WD40 modules contain the binding site for late endosomes/lysosomes and the DH-like (DH_L_)-pleckstrin homology (PH) domains is the lipid droplet-binding region of VPS13C [22]. We showed published PTC mutations and deletions below the VPS13C protein, with at the left first author name and year of publication [11, 24, 37]

Short-read paired-end WGS of two siblings affected with DLB (family A, Fig. 1a), subsequent read alignment to the human reference genome (GRCh37/hg19) and base and variant calling were performed by Complete Genomics™ Inc [13]. Targeted resequencing of all 86 coding exons and flanking splice sites of *VPS13C* was performed using amplicon-target PCR amplification (MASTR technology; Agilent), followed by sequencing on the MiSeq platform (Illumina). Sanger sequencing was used to analyze *VPS13C* exons 7–8, 27, 37–38, 41, 46, 54, 60–61, 70–73, 76–77 and 80, which were < 85% 20X covered with the MASTR assay, and to validate and genotype rare (minor allele frequency (MAF) ≤ 1%) coding and splice site variants with a potential impact on the protein sequence identified by targeted resequencing in patients and family members of whom DNA was available. Compound heterozygous variants were phased in order to identify carriers with *trans* configuration of *VPS13C* variants. Short tandem repeat (STR) markers were used to identify shared alleles and to determine haplotypes in individuals sharing rare coding or splice site variants in *VPS13C.*

### *VPS13C* mRNA and protein analysis

The effect of splice-site variants on mRNA splicing was analyzed in silico and in cultured lymphoblast cells, if available, for carriers of compound heterozygous coding and splice-site mutations in *VPS13C*. For *VPS13C* mRNA expression analysis via quantitative RT-PCR, total RNA was isolated from cultured lymphoblast cells of family A and 3 unrelated control individuals. Western blotting, to evaluate the effect of coding and splice site variants on VPS13C protein expression, was performed on protein lysates of lymphoblast cells, if available, of patient P1 and the unaffected parents of family A, homozygous and compound heterozygous carries and of unrelated control individuals negative for rare *VPS13C* variants (MAF ≤ 1%). Also, VPS13C protein expression was evaluated via Western blotting in brain tissue of patients P1 and P3 with *trans* compound heterozygous *VPS13C* missense mutations and 2 unrelated control individuals negative for rare *VPS13C* variants (MAF ≤ 1%).

### Subcellular localization of VPS13C

cDNA constructs containing the coding sequence of wild type (WT) or mutant (p.Trp395Cys or p.Ala444Pro) human *VPS13C* were transfected in HeLa or SH-SY5Y cells to perform immunohistochemistry and live cell labeling.

### Neuropathology

Autopsy brains of DLB patients P1 (family A) and P3 (family B) with *trans* compound heterozygous VPS13C missense mutations were fixed in formalin for 13 weeks and 5 weeks, respectively. Samples, taken from the frontal cortex areas 4, 6, 8, 9, 10, 11, 12, 24 and 46, the superior temporal cortex, hippocampus and amygdala, parietal and occipital cortex, the thalamus, neostriatum, pallidum, mesencephalon, pons, medulla oblongata and cerebellum. Cytological stains included Cresyl-Violet, Hematoxylin–Eosin, and Klüver-Barrera as myelin stain. Immunohistochemistry, performed with antibodies against β-amyloid (4G8), hyperphosphorylated tau (AT8), ubiquitin, TDP-43, FUS, p62 and α-synuclein.

### Statistics

Burden and variance-component tests implemented in the optimized sequence kernel association test (SKAT-O) provided in the R package SKAT v2.0.0, used to investigate association of single *VPS13C* variants with MAF ≤ 1% and PD. First, power calculation was performed within the SKAT framework using a logistic test for dichotomous traits (target sequence: 12,941 bp, causal variant percentage = 20%, negative variant percentage = 20%, Maximal OR = 5). Under these conditions, the total sample cohort required to reach 80% power with a 0.05 significance level is at least 1050 individuals. Our patient and control cohorts consist of 1508 individuals and met the requirements. Adjustment to SKAT-O applied taken the small sample size (< 2000 individuals). Gender was included as covariate. We considered a two-sided *p* value < 0.05 significant. To investigate association between bi-allelic *VPS13C* variants and LBD, we compared statistically the variant frequencies between the patient and control group using Fisher’s exact statistics. Data are represented as the average ± standard deviation of a minimum of 3 independent experiments. For the description of the statistical significance of differences, the Multiple Comparisons of a one-way ANOVA using the GraphPad Prism V7.01 software calculated P-values. Values were considered significant if * 0.01 < *P* < 0.05; **0.001 < *P* < 0.01; *** 0.0001 < *P* < 0.001; ****P < 0.0001.

## Results

### Clinical phenotype of family A

In family A (Fig. 1a), genomic DNA was available of two affected siblings and their unaffected parents. The index patient, P1 (II.2. Fig. 1a), developed clinical symptoms at age 42. Soon after, language problems occurred, particularly word finding difficulties, dominating the clinical picture for several years. At the age of 47, a clinical neurological examination revealed non-fluent aphasia, extrapyramidal signs consisting of hypomania, bradykinesia, gait disturbances, cogwheel rigidity and resting tremor (Additional file 1: Table S2). Frontal disinhibition signs such as glabella reflex, snout reflex and palmomental reflexes were also present. Later in the disease course, myoclonus was observed, and behavioral symptoms became more apparent i.e. social withdrawing, passivity and changed taste preferences. An early brain magnetic resonance imaging (MRI) scan showed moderate bilateral prefrontal atrophy, and later in the disease, also temporal and biparietal atrophy. Single-photon emission computed tomography (SPECT) imaging in the initial phase of the disease was compatible with AD diagnosis, however, AD pathology was not confirmed by cerebrospinal fluid (CSF) biomarker analysis (Additional file 1: Table S2). The disease progressed rapidly, and the patient died at age 54. Brain autopsy of patient P1 neuropathology confirmed diffuse LBD, neocortical type. Patient 2, the affected sibling of patient P1 (II.1, Fig. 1a), presented at age 41 with initial symptoms of anxiety and depression, combined with word finding difficulties. An episode of visual hallucinations and delusions was reported when the first symptoms appeared. Clinical neurological examination at age 43 revealed frontal disinhibition signs, compromising a glabella reflex and a positive snout reflex, the occurrence of myoclonus, extrapyramidal signs of cog-wheel rigidity and dysarthria, perseveration, semantic paraphasias and constructional apraxia. Later in the disease course, an ataxic finger-to-nose test was observed and symptoms of apathy, loss of initiative and episodic memory problems were reported. A brain MRI early in the disease showed frontal atrophy whereas a brain fluorodeoxyglucose positron emission tomography (FDG-PET) scan showed hypometabolism of the left frontal cortex and both temporal and parietal lobes. Similar to patient P1, CSF biomarker levels were not suggestive for AD pathology. Patient P2 received a clinical diagnosis of unspecified dementia and died at the age 47. There were no other familial antecedents of early-onset neurodegenerative brain disease. Both parents were unaffected at advanced ages (> 80 years), showing that segregation of the disease in family A is consistent with autosomal recessive inheritance (Fig. 1a).

### Identification of compound heterozygous *VPS13C* variants in family A

We obtained WGS data of the two affected siblings of family A (Fig. 1a). Variant filtering resulted in 178 rare (MAF < 1%) coding variants with an impact on protein level and splice site variants, shared by the affected siblings. None of these coding variants were shared homozygous by the affected siblings. In four genes, Cellular Communication Network Factor 6 gene (*CCN6*), Ring Finger Protein 6 gene (*RNF6*), Chronic Lymphocytic Leukemia Up-regulated 1 gene (*CLLU1)* and the Vacuolar Protein Sorting 13 homolog C gene (*VPS13C*), we observed compound heterozygous coding variants shared by the affected siblings, including 8 missense mutations and 1 PTC mutation (Additional file 1: Table S3). Only in *VPS13C*, the compound heterozygous missense mutations, p.Trp395Cys and p.Ala444Pro were present in the unaffected parents carrying each one the missense mutation. This observation confirms that the compound heterozygous missense mutations in *VPS13C* are located in *trans* in the affected siblings and were inherited in a recessive pattern (Fig. 1a). Also, the *VPS13C* p.Trp395Cys and p.Ala444Pro mutations were absent in the control cohort and belong to the 1% most deleterious amino acid substitutions in the human genome, indicated by a CADD_Phred score above 20 (Table 1) [21]. The multiple heterozygous variants in *CCN6*, *RNF6* and *CLLU1* were all located in *cis*, present in one parent and absent in the other parent of family A.

**Table 1:** LBD patients carrying rare homozygous or compound heterozygous mutations in *VPS13C*

Patient	Dx	AAO	∆CDS^a^	ΔAA^b^	MAF gnomAD_nfe (%)	CADD_Phred score^c^	MAF patient cohort (%) n = 844	MAF control cohort (%) n = 664	Phase	F in patients (%)	F in controls (%)	F expected^d^ (%)
P1*^,$^	DLB	42	c.1185G > C	p.Trp395Cys	–	33	0.0592	0	*Trans*	0.118	0	0.0000770
			c.1330G > C	p.Ala444Pro	0.00864	28.4	0.415	0				
P2*	DLB	40	c.1330G > C	p.Ala444Pro	0.00864	28.4	0.415	0	Homozygous	0.118	0	0.0005
			c.1330G > C	p.Ala444Pro	0.00864	28.4	0.415	0				
P3*^,$^	DLB	58	c.3652A > G	p.Thr1218Ala	0.000781	26.4	0.0592	0	*Trans*	0.118	0	0.00020
			c.8366 T > C	p.Ile2789Thr	1.00	2.797	0.533	0.679				
P4	DLB	84	c.8133G > A	p.Met2711Ile	0.00177	22.5	0.0592	0.0754	*Trans*	0.118	0	0.000396
			c.8366 T > C	p.Ile2789Thr	1.00	2.797	0.533	0.679				
P5*	PD	60	c.5060C > T	p.Ala1687Val	0.314	22.5	0.0592	0.151	*Trans*	0.118	0	0.000264
			c.8711C > T	p.Ser2904Leu	0.343	26.7	0.178	0.378				
P6*	DLB	42	c.2797A > G	p.Thr933Ala	0.577	12.67	0.711	0.452	Unknown	0.118	0	0.000991
			c.7276G > A	p.Val2426Ile	0.244	12.31	0.118	0.226				
7*	PD	65	c.2296A > G	p.Thr766Ala	–	12.18	0.0592	0.0754	Unknown	0.118	0	0.0000220
			c.5537 T > C	p.Leu1846Ser	–	26.8	0.0592	0				
P8	PD	73	c.4056 + 3A > C	–	0.00705	14.06	0.0592	0	Unknown	0.118	0	0.0000110
			c.7628C > G	p.Ser2543Cys	–	28.9	0.0592	0				
P9	PD	73	c.8366 T > C	p.Ile2789Thr	1.00	2.797	0.533	0.679	Unknown	0.118	0	0.00337
			c.11176A > G	p.Ile3726Val	0.594	23.1	0.237	0.980				

Clinical symptoms of LBD patients with (putative) recessive inherited mutations are summarized in Additional file 1: Table S2

*Dx* diagnosis, *PD* Parkinson’s disease, *DLB* Dementia with Lewy bodies, *∆CDS* coding sequence substitution, *ΔAA* amino acid substitution, *MAF* minor allele frequency, *F* frequency of homozygotes and compound heterozygotes, *AAO* age at onset, *gnomAD_nfe* Genome Aggregation Database non-Finnish European population [23]

^*^Lymphoblast cells available

^$^brain tissue available

^a^Coding nomenclature according to NM_020821

^b^Protein nomenclature according to NP_0658721

^c^CADD_Phred score [21]

^d^ Expected frequency is calculated according to the Hardy–Weinberg principle, using the MAF of the single alleles in patients plus controls (n = 1508)

### Gene-based targeted resequencing of *VPS13C* in the Belgian LBD patient and control cohorts

In the 86 coding exons and splice sites of *VPS13C*, we identified 71 rare (MAF ≤ 1%) variants with a potential impact on the protein sequence in the LBD patient and control cohorts: 64 missense mutations, 1 nonsense mutation and 6 splice site variants (Additional file 1: Table S4, Fig. S1). All *VPS13C* variants were present in isoform 2, the largest *VPS13C* transcript (NM_020821.2), containing exons 6 and 7, and the main splice variant in brain, suggesting brain-specific gene functions [42]. In the Belgian cohort, 86 LBD patients (86/844, 10.2%), including 22 DLB (22/233, 9.44%) and 64 PD (64/611, 10.47%) patients, carried a rare variant in *VPS13C*, compared to 86 control individuals (13.0%). We performed a SKAT-O analysis on the 71 rare variants and observed a significant association (*p* = 0.0233) between rare coding (impact on protein sequence) and splice site *VPS13C* variants and LBD (Additional file 1: Table S4).

### Investigation of a potential pathogenic role of the homozygous and compound heterozygous missense mutations

We focused on patients and controls carrying homozygous or compound heterozygous, rare missense or splice site variants in *VPS13C* obtained in the targeted resequencing data. Detailed results on phasing are available in the Supplementary Results. Besides patient P1 (Family A, Fig. 1a), we identified 3 additional patients with *trans* compound heterozygous missense mutations, 1 patient with homozygous missense mutations and 3 patients with compound heterozygous missense mutations of unknown phase in *VPS13C* (Table 1). The frequency of patient carriers with (putative) autosomal recessive inherited *VPS13C* mutations, including the 4 non-phased patient carriers is 1.07% (9/844). All their compound mutated alleles are clustering in VPS13α domain involved in lipid transport, the putative WD40 domain involved in late endosomal/lysosomal localization or the pleckstrin homology domain involved in lipid droplet binding (Fig. 1b) [22]. The clinical characteristics of all 9 patient carriers are summarized in Additional file 1: Table S2. All were negative for mutations in the major PD genes and other genes associated with neurodegenerative brain diseases (Additional file 1: Table S5), except for the PD patient P9 who carried the *LRRK2* p.Arg1441Cys pathogenic mutation besides the *VPS13C* p.Ile2789Thr/p.Ile3726Val un-phased alleles. *Cis* compound heterozygous missense or splice site variants in *VPS13C* were identified in 3 patients (Additional file 1: Table S6). Of the 7 compound heterozygous carriers in the control group, 5 control individuals (C3, C4, C5, C6 and C7) have a confirmed *cis* configuration of their *VPS13C* missense or splice site variants while the 2 remaining controls had no confirmed phasing (2/664; 0.30%; Additional file 1: Table S7). The observation of 5 LBD carriers (5/844; 0.59%) versus zero control carriers (0/664; 0%) of homozygous and *trans* compound heterozygous mutations in line with recessive inheritance is suggestive of an enrichment in patients, though not significant (*p* = 0.071).

### Effect of homozygous and compound heterozygous mutations on VPS13C expression

**Fig. 2:**
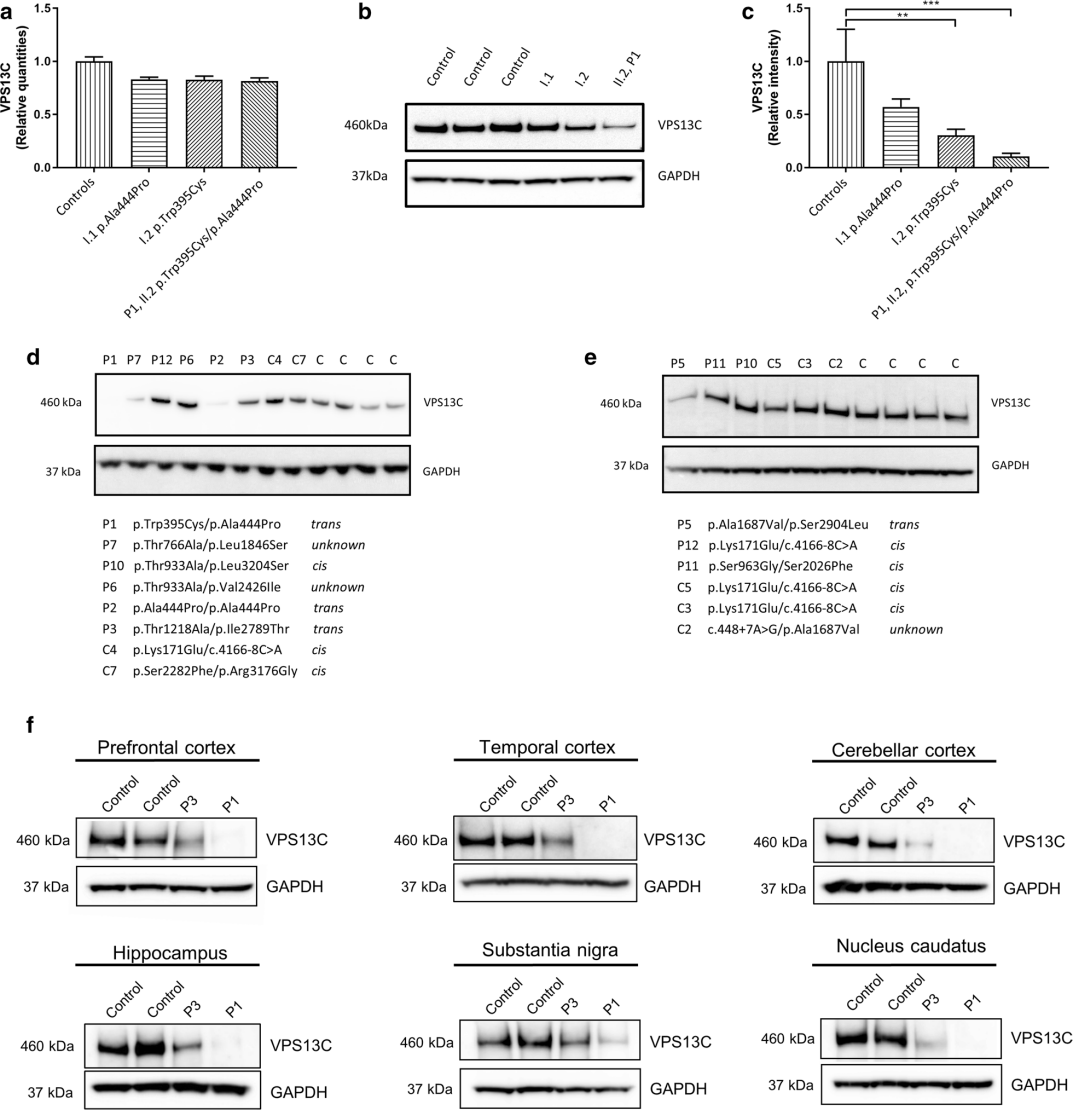
Reduced VPS13C protein expression in lymphoblast cells and brain lysates of mutation carriers. **a** Relative *VPS13C* mRNA expression levels of family A and unrelated control individuals (n = 3) in lymphoblast cells. **b** Representative immunoblots of endogenous VPS13C expression in family A and unrelated control individuals (n = 3) in lymphoblast cells. **c** Quantification of VPS13C protein expression, normalized with the expression of GAPDH in lymphoblast cells. Error bars represent standard deviation. **d–e** Representative immunoblots of endogenous VPS13C protein expression in lymphoblast cells of patient and control carriers with homozygous or compound heterozygous mutations in *VPS13C*, and unrelated control individuals (n = 4). **f** Representative immunoblots of endogenous VPS13C protein expression in brains of patient carrier P1 and P3 and two unrelated control individuals. Protein levels were measured in the prefrontal cortex (**d**), the temporal cortex (**e**), the cerebellar cortex (**f**), the hippocampus (**g**), the substantia nigra (**h**), the caudate nucleus (**i**) and the putamen (**j**); **0.001 < *P* < 0.01; *** 0.0001 < *P* < 0.001; ****P < 0.0001

Results of *VPS13C* splice site variants in compound heterozygous (unkown phase and *cis*) carriers (Table 1, Additional file 1: Table S6, Table S7) on mRNA splicing are available in the Supplementary Results. Briefly, we did not observe exon skipping of c.4166-8C > A in lymphoblast cells, c.4056 + 3A > C was predicted to affect the canonical splice donor site of exon 36 and the in silico results of c.448 + 7A > G were inconsistent between prediction programs. To determine the effect of the *VPS13C* missense mutations, p.Trp395Cys and p.Ala444Pro, identified in family A on transcript and protein expression, we used qPCR and Western blot analysis of lymphoblast cells of DLB patient P1 (Fig. 1a, II.2) and unaffected parents (Fig. 2a–c). No lymphoblast cells were available of the affected sibling of P1 (Fig. 1a, II.1). We did not observe significant difference in *VPS13C* transcript expression for patient P1 and parents, compared to controls negative for rare *VPS13C* variants (Fig. 2a). However, endogenous VPS13C protein expression was reduced ~ 40% in the parent carrying the p.Ala444Pro mutation (*p* = 0.0618) and ~ 70% in the parent carrying the p.Trp395Cys mutation (*p* = 0.0024). In the patient P1, carrying both *VPS13C* mutations p.Trp395Cys/p.Ala444Pro, there is 90% reduction (*p* = 0.0002), compared to the control individuals (Fig. 2b, c). We further investigated VPS13C protein expression in all patient and control carriers of homozygous or compound heterozygous (*trans*, unknown phase and *cis*) *VPS13C* variants with lymphoblast cells available. In patient P2, homozygous for the p.Ala444Pro mutation, the severe reduction in VPS13C protein expression was comparable to the expression level observed in patient P1, *trans* compound heterozygous for p.Trp395Cys/p.Ala444Pro (Fig. 2d). Moreover, we observed a reduced expression in patient P5, *trans* compound heterozygous for p.Ala1687Val/p.Ser2904Leu and patient P7 un-phased compound heterozygous for p.Thr766Ala/p.Leu1846Ser (Fig. 2d, e). None of the control carriers and the *cis* compound heterozygous carriers showed a reduction in VPS13C protein expression (Fig. 2d, e). Brain tissue was available of patients P1 and P3, both with *trans* compound heterozygous *VPS13C* missense mutations, and two unrelated control individuals negative for rare *VPS13C* variants (MAF ≤ 1%). In all studied brain regions (*prefrontal cortex*, *temporal cortex*, *cerebellar cortex*, *hippocampus*, *substantia nigra* and *nucleus caudatus*), VPS13C protein expression was abnormally reduced in patients P1 and P3 compared to control individuals, with almost no VPS13C protein levels in patient P1 (Fig. 2f) of Family A, the discovery family we used for gene identification (Fig. 1a).

### Subcellular localization of VPS13C

**Fig. 3:**
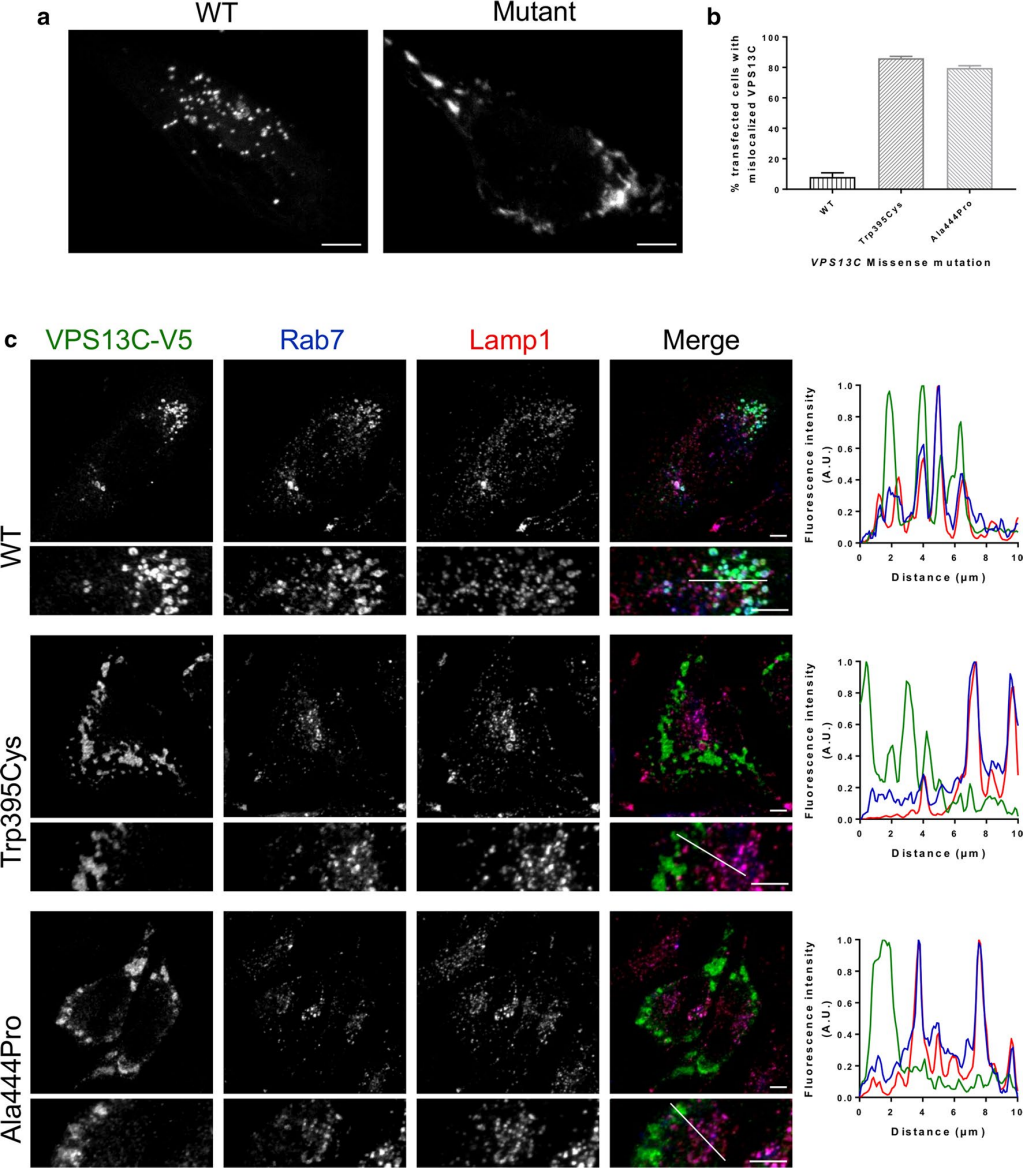
Localization of VPS13C at the late endosomes and lysosomes disturbed by p.Trp395Cys and p.Ala444Pro. Triple immunofluorescence of V5 (VPS13C constructs, green) with markers for late endosomes (Rab7) and lysosomes (Lamp1) in HeLa cells. **a** Cells expressing VPS13C WT showed a clear vesicular staining pattern while those expressing the missense mutations p.Trp395Cys or p.Ala444Pro mislocalized in larger cytosolic structures. **b** Quantification of cells that appeared either vesicular or mislocalized. **c** Staining with anti-V5 antibody in WT-overexpressing HeLa cells showed co-staining with markers against late endosomes and lysosomes. HeLa cells overexpressing either p.Trp395Cys or p.Ala444Pro showed no co-localization with endosomes and lysosomes. White lines indicate the position of the intensity profiles. Scale bars = 5 µm

We transfected HeLa cells with wild type or mutant (p.Trp395Cys or p.Ala444Pro) VPS13C and investigated the effect of the missense mutations on the subcellular localization of the protein. Wild type VPS13C localized to small organelles, whereas mutant VPS13C alleles, p.Trp395Cys or p.Ala444Pro, localized at larger cytosolic structures in most cells (Fig. 3a-b). Triple immunostaining of V5 (VPS13C constructs with a C-terminal V5-6 × His tag), with markers for late endosomes (Rab7) and lysosomes (Lamp1), revealed a late endosomal/lysosomal localization of wild type VPS13C. However, the late endosomal/lysosomal localization of VPS13C was lost when the VPS13C mutant alleles were present (Fig. 3c), which was independently confirmed with GFP-tagged VPS13C constructs and other markers (CD63, late endosomes; Lysotracker, lysosome; Additional file 1: Fig. S2, S3). Moreover, we could confirm this subcellular localization of wild type and mislocalization of mutant VPS13C at late endosomes and lysosomes in human neuroblastoma SH-SY5Y cells (Additional file 1: Fig. S4). Immunostaining in HeLa cells with markers for the ER (PDI; Additional file 1: Fig. S5), cis- and medial-Golgi (Giantin; Additional file 1: Fig. S6) and trans-Golgi (TGN46; Additional file 1: Fig. S7) demonstrated no co-localization of wild type and mutant VPS13C with these organelles.

### Pathological phenotype of compound heterozygous missense mutation carriers

**Fig. 4:**
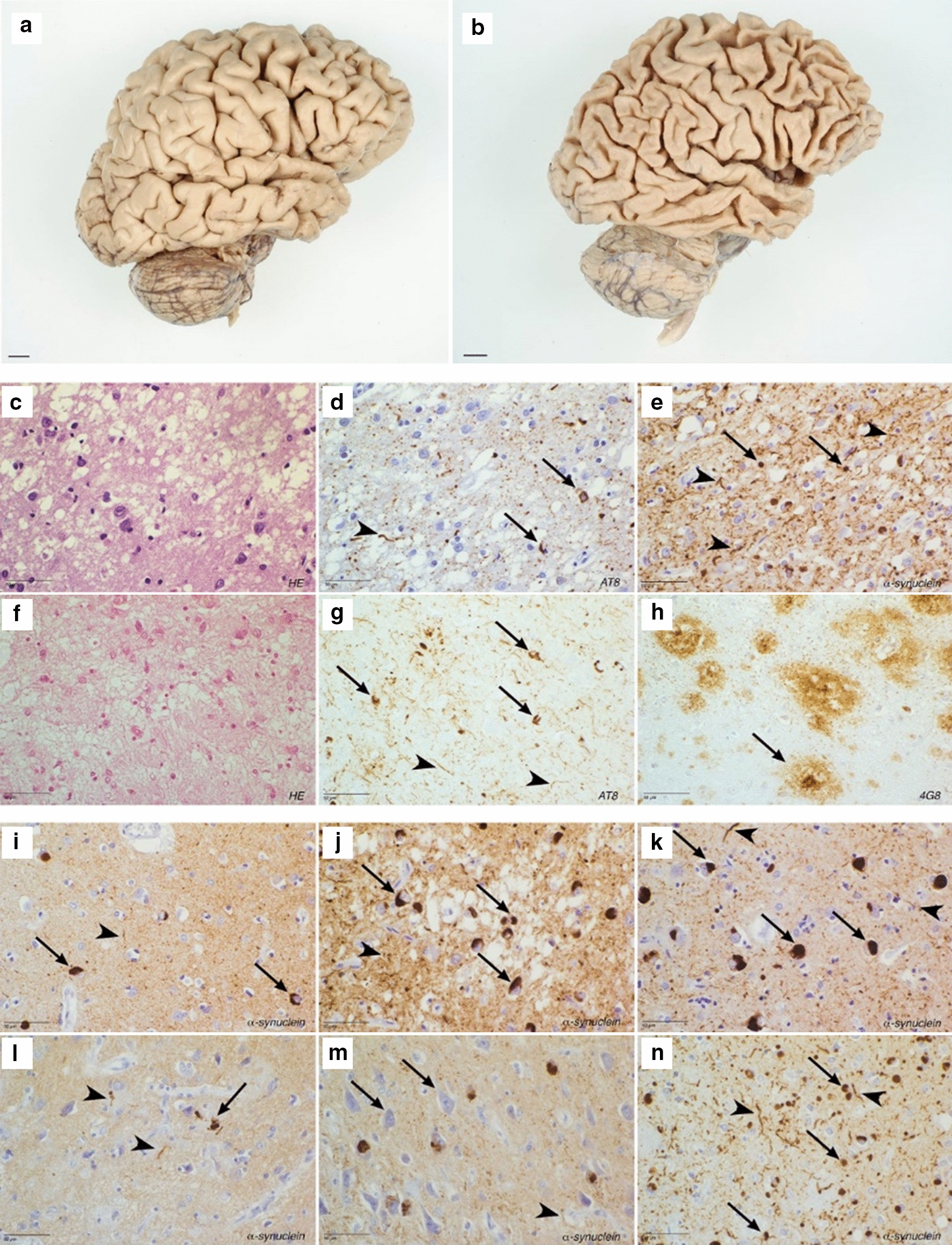
Neuropathology of patient carriers P1 and P3. **a** Right hemisphere of patient P1 shows moderate frontotemporal atrophy, with the superior temporal gyrus more affected than the medial temporal gyrus. **b** Right hemisphere of patient P3 shows severe cortical atrophy, more pronounced in the frontal and temporal lobes. The pre- and post-central gyrus are relatively spared. **c–e** The lateral occipitotemporal gyrus of patient P1 shows (**c**) microspongiotic changes in the cortex (Hematoxylin–Eosin stain), (**d**) a relatively mild load of neurofibrillary tangles (arrow) and neuritic threads (arrowhead) (AT8 stain), and (**e**) severe α-synuclein pathology with Lewy bodies (arrow) and Lewy neurites (arrow head). **f–h** The frontal cortex of patient P3 shows (**f**) severe microspongiosis and neuronal loss (Hematoxylin–Eosin stain), (**g**) the abundance of hyperphosphorylated tau pathology with many neurofibrillary tangles (arrow) and neuritic threads (arrowhead) (AT8 stain), and (**h**) severe β-amyloid pathology with many classic (arrow) and diffuse senile plaques (4G8 stain). **i–k** α-synuclein pathology of patient P1 in (**i**) the frontal cortex, (**j**) the hippocampus (severe) and (**k**) the amygdala (severe). **l–n** α-synuclein pathology of patient P3 in (**l**) the frontal cortex, (**m**) the hippocampus (moderate) and (**n**) the amygdala (severe). Lewy bodies are marked with arrows, Lewy neurites with arrowheads

Patient P1 died at age 54, and we obtained the autopsy brain with 12 h postmortem delay (PMD). We observed moderate frontotemporal atrophy with the superior temporal gyrus affected more than the medial temporal gyrus (Fig. 4a). In the midbrain, the zona compacta of the substantia nigra was very pale. The substantia nigra showed severe neuronal loss, most explicit in the lateral part of the zona compacta. Using the rating scheme for cerebrovascular lesions of Deramecourt and colleagues, no more than grade 2 of vascular pathological alterations could be scored [12]. The lateral occipitotemporal gyrus of patient P1 showed microspongiotic changes in the cortex (Fig. 4c). The hippocampus and parahippocampal gyrus were affected with a moderate number of neurofibrillary tangles, neuritic threads and dystrophic neurites (Fig. 4d), whereas other brain structures did not present tau pathology. 4G8 staining to detect β-amyloid pathology and TDP-43 and FUS staining for frontotemporal dementia (FTD) pathology were all negative. The α-synuclein staining showed an abundance (grade 3) of Lewy bodies and Lewy neurites in the frontal cortices, temporal neocortex, hippocampus, parahippocampal gyrus, amygdala, and in the pigmented nuclei of the mesencephalon, pons and medulla oblongata (Fig. 4e, i–k). Rare Lewy body pathology was found in the occipital cortex, neostriatum, and hypothalamus. Based on our neuropathological findings, the patient received a diagnosis of diffuse Lewy body disease, neocortical type [20, 28]. Patient P3 died at age 64 and autopsy brain was obtained 8 h following death. We observed severe atrophy of the temporal lobe while the parietal and occipital lobes were less severely affected (Fig. 4b). Ventricular dilation was severe and most explicit in the temporal horn. The pars compacta of the substantia nigra was markedly thin and more rostrally, completely depigmented. The locus caeruleus in the pons was also severely depigmented. Histochemistry showed severe neuronal loss in the frontal and temporal cortices, and to a lesser extent in the parietal cortex with spongiosis and astrocytic gliosis (Fig. 4f). There was a severe atrophy of the hippocampus, amygdala and parahippocampal gyrus. The superior temporal gyrus was severely gliotic, with a thinning of the cortex to 1.5 mm. The dorsomedial formation of the thalamus was affected with neuronal cell loss and gliotic changes and severe neuronal loss was observed in the substantia nigra. Neurofibrillary tangles, neuropil threads and dystrophic neurites is found in every cortical sample examined, as well as the thalamus, neostriatum, corpus mamillare, medial geniculate body, substantia nigra, and reticular formation of mesencephalon, pons and medulla oblongata (Fig. 4g). The lesion load was compatible with that of stage VI (Braak and Braak) [7], and with a stage B3 of Montine et al. [20]. Classical and diffuse senile plaques were present in frontal, temporal, parietal and striatal cortices, as well as in the thalamus, putamen, medial geniculate body, corpus mamillare and in the molecular layer of the cerebellar cortex (Fig. 4h). Cerebral amyloid angiopathy was mild [25] and the β-amyloid pathology was compatible with Phase 5 of Thal et al. [39], whereas the load of classical senile plaques was severe, compatible with CERAD stage 3 [30]. These findings are compatible with AD neuropathological changes A3B3C3 [20]. Both the TDP-43 and FUS stainings were negative. α-synuclein staining showed a moderate amount of Lewy bodies in the hippocampus and parahippocampal gyrus, and a severe amount in the amygdala. Sparse Lewy bodies were found in prefrontal cortex, the substantia nigra, the pons and the medulla oblongata (Fig. 4l–m). Based on these observations, patient P3 received a neuropathological diagnosis of LBD, amygdala predominant type, and of AD neuropathological changes A3B3C3 [20, 28].

## Discussion

A risk variant in the *VPS13C* locus was genome-wide significant in a meta-analysis of genome-wide association studies (GWAS) with an estimated odds ratio of 1.1 [10, 31, 32]. Lesage and colleagues, identified homozygous and compound heterozygous PTC mutations in *VPS13C* in patients with a distinct form of early-onset parkinsonism, characterized by rapid and severe disease progression and early cognitive decline (Fig. 1b) [24]. The presence of Lewy bodies in the brainstem, limbic system and many cortical areas in one of the PD patients was reminiscent of diffuse LBD [24]. Later, diagnostic whole exome sequencing of 80 early-onset PD patients identified compound heterozygous variants affecting canonical splice sites, leading to PTCs, in *VPS13C* in one isolated early-onset PD patient (Fig. 1b) [37]. This patient presented overall with milder motor symptoms and disease progression, but with a rapid deterioration of cognitive functioning [37]. Shortly after, a large homozygous deletion of 50 exons of *VPS13C,* was identified by WGS in a sporadic patient with sensorimotor polyneuropathy and early-onset parkinsonism (Fig. 1b) [11]. This patient presented with normal cognitive functioning and a milder disease severity. In a Chinese early-onset PD cohort of 669 patients, 7 isolated patients (1.05%) were identified with rare compound heterozygous missense, nonsense and splice site variants in *VPS13C*, of which 4 patients with confirmed *trans* compound heterozygous variants [17]. Taken together, autosomal recessive LOF mutations in *VPS13C* are rare and associated with early-onset PD, with a high probability of cognitive deterioration and suggestive diffuse LBD pathology. However, the impact *VPS13C* missense mutations on protein expression and functioning, and the contribution of *VPS13C* missense mutations to disease etiology was not yet known. In our study, we identified *trans* compound heterozygous missense mutations, p.Trp395Cys and p.Ala444Pro, reducing VPS13C protein expression in family A affected with autosomal recessive early-onset and pathologically confirmed DLB (Fig. 1a). Our findings in family A triggered our interest in the role of rare missense mutations in *VPS13C* and risk for LBD. Targeted resequencing of *VPS13C* in the Belgian LBD patient and control cohorts, identified a significant association (*p* = 0.0233) between rare (MAF ≤ 1%) coding variants with an impact on the protein sequence (n = 65) and splice site (n = 6) variants in *VPS13C* and LBD. A full burden test (rho = 1) was not significant (*p* = 0.175), indicating a complex architecture of pathogenic, protective and benign variants in *VPS13C*. Interestingly, targeted resequencing of *VPS13C* in 1567 late-onset PD patients identified a haplotype, including the common (MAF > 1%) coding *VPS13C* variants p.Arg153His-p.Ile398Ile-p.Ile1132Val-p.Gln2376Gln, contributing to reduced PD risk (*p* = 0.0052, odds ratio = 0.48, 95% confidence interval = 0.28–0.82) [36]. Another independent study, investigating 4476 sporadic PD patients (mean AAO 60 years) and 5140 healthy control individuals, reported a significant association (*p* = 0.002296) of rare (MAF ≤ 1%) *VPS13C* variants in PD [19].

We identified in the Belgian LBD cohort, in addition to patient P1 (Family A, Fig. 1a), 3 patients (P3, P4 and P5) with recessive compound heterozygous missense mutations, 1 patient (P2) with homozygous missense mutations and 4 patients (P6, P7, P8 and P9) with compound heterozygous mutations of unknown phase in *VPS13C* (Table 1). The frequency of LBD patient carriers of proven recessive compound heterozygous missense mutations is 0.59% (5/844). In the control cohort, we observed two carriers of compound heterozygous rare coding and splice site variants in *VPS13C* of unknown phase (Additional file 1: Table S7). These observations are suggestive for an enrichment of recessive inherited *VPS13C* mutations in patients compared to control individuals. However, the statistical analysis was not significant (*p* = 0.071), most likely because of the small numbers in the Belgian cohorts and the inability to phase all mutations in patients and control individuals.

Limitations in *cis*/*trans* phasing of compound heterozygous mutations complicate the genetic evidence for autosomal recessive genes. One inherent limitation of short-read sequencing technologies is that the phase of long distance variants is lost in the sequencing reads. We used different methodologies to overcome this hurdle, including genotyping relatives, haplotype sharing analysis, allele-specific PCR for short distance mutations and Oxford Nanopore Technologies long-read cDNA sequencing for long-distance mutations, but limitations remain. Due to the long genomic distance of many *VPS13C* mutations, long-read sequencing could only be performed on cDNA level. Therefore, without the availability of biomaterials for RNA isolation of the desired isoform, or DNA of relatives for genotyping, phasing of long distance *VPS13C* mutations was practically impossible. Additionally, long-read sequencing technologies are less accurate in the detection of single nucleotide variants compared to short-read sequencing technologies. Consequenly, not all missense mutations in carriers could be called in long-read sequencing data (Additional file 1). Replication of rare PTC and missense mutations in *VPS13C* in independent and larger cohorts is necessary, but optimized methods are also urgently needed to confirm *trans* configuration of compound heterozygous mutations. Only then we are able to estimate the contribution of recessive inherited *VPS13C* mutations to LBD etiology.

DLB patients P1 with compound heterozygous mutations p.Trp395Cys/p.Ala444Pro, P2 with homozygous p.Ala444Pro and P3 with p.Thr1218Ala/p.Ile2789Thr presented with a severe disease progression (Additional file 1: Table S2). Neuropathological examination of patient carriers P1 and P3 indicated Lewy bodies in multiple brain regions (Fig. 4i–n). Since there was also extensive AD pathology in patient P3, there is a small likelihood that the pathological findings are associated with a DLB clinical syndrome. Nevertheless, both our clinical and neuropathological data of *VPS13C* mutation carriers supports the phenotype of most *VPS13C* patient carriers reported to date, including an early-onset age, severe disease progression and the co-occurrence of parkinsonism and dementia (Additional file 1: Table S2) [11, 24, 37]. The variability in onset age and the presence of potential compound heterozygotes in the control group might be explained by variable penetrance and variable loss of protein expression and functioning of the *VPS13C* mutant missense alleles. In the patients P1 (family A) and P2, with bi-allelic p.Trp395Cys and/or p.Ala444Pro, the reduction of VPS13C protein expression in lymphoblast cells was the most severe (Table 1, Fig. 2d). Both patients developed the disease at very early age (40–42 years) and had a marked severe disease progression (Additional file 1: Table S2). In brain tissue of the two patients, P1 and P3 (family B), with confirmed *trans* compound heterozygous *VPS13C* missense mutations, VPS13C protein expression was remarkably reduced compared to control individuals, with the strongest reduction observed for patient P1 (Fig. 2f). The *VPS13C* missense mutations of DLB patient P3 were present in one younger sibling II.4 (Family B; Fig. 1a). This sibling’s age 62 is close to the onset age of the index patient P3 indicating that this sibling is still at risk for disease.

Research showed that human VPS13C functions as a tether between the ER and late endosomes/lysosomes, and between the ER and lipid droplets, enabling transport of glycerolipids between membranes [22]. We confirmed the localization of wild type VPS13C at late endosomes and lysosomes (Fig. 3, Additional file 1: Fig. S2, Fig. S3, Fig. S4). Overexpressing wild type or mutant VPS13C, containing p.Trp395Cys or p.Ala444Pro, in HeLa and SH-SY5Y cells demonstrated that the late endosomal/lysosomal localization of VPS13C is completely lost in mutants (Fig. 3, Additional file 1: Fig. S2, Fig. S3, Fig. S4). Surprisingly, both mutations are in the VPS13α domain and not in the putative WD40 modules responsible for endosomal/lysosomal localization (Fig. 1b). Because these mutations are nearby the FFAT-motif required for interaction with the ER, they may directly affect the localization to ER-contact sites as well. Overall, the different domains might be required for the protein’s structural stability, needed for its localization to ER-late endosome/lysosome and ER-lipid droplet contact sites. Mutations within the VPS13α domain may overall negatively affect the stability of the protein thereby affecting its localization. Besides p.Trp395Cys or p.Ala444Pro, we identified 12 alleles in patient carriers of (putative) recessive inherited *VPS13C* mutations, including 11 missense and one splice site mutations (Fig. 1b; Table 1), awaiting further functional investigation to estimate their pathogenicity.

## Conclusions

Overall, our results suggest that rare homozygous and compound heterozygous missense mutations in *VPS13C* contribute to both PD and DLB risk. We identified *trans* compound heterozygous missense mutations p.Trp395Cys and p.Ala444Pro in *VPS13C* with loss of functional protein, confirming their pathogenicity. Understanding the contribution of the different mutated *VPS13C* alleles to the genetic etiology of LBD needs additional research.

## Supplementary Information

**Additional file 1**. Supplementary materials and methods. Detailed protocols and references. Supplementary results. Phasing compound heterozygous variants in the Belgian cohorts. Effect of VPS13C splice site variants on mRNA splicing. Supplementary tables. **Table S1**: Clinical and demographic characteristics of study cohorts. **Table S2**. Clinical data of patient carriers of rare homozygous or *trans *compound heterozygous *VPS13C *mutations. **Table S3**: Candidate genes and mutations identified in WGS data of family A. **Table S4**: *VPS13C* rare coding and splice site variants in DLB and PD patients and in controls. **Table S5**: Major genes associated with neurodegenerative brain diseases. **Table S6**: LBD patient carriers of *cis *compound heterozygous coding and splice site variants in *VPS13C*. **Table S7**: Control carriers of compound heterozygous coding and splice site variants in *VPS13C*. **Table S8**: Primer sequences. **Table S9**: *In-silico* predictions on *VPS13C* mRNA splicing of splice site variants in compound heterozygous carriers. Supplementary figures. **Fig. S1**: *VPS13C *rare coding and splice site variants in DLB (n=233) and PD patients (n=611), and in controls (844). **Fig. S2**: Late endosomal localization of VPS13C is lost in p.Trp395Cys and p.Ala444Pro mutations. **Fig. S3**: Lysosomal localization of VPS13C is lost in p.Trp395Cys and p.Ala444Pro mutations. **Fig. S4**: Missense mutations p.Trp395Cys and p.Ala444Pro disturb localization of VPS13C at the lysosomes. **Fig. S5**: Wild type and p.Trp395Cys or p.Ala444Pro mutant VPS13C does not accumulate at the endoplasmic reticulum. **Fig. S6**: Wild type and p.Trp395Cys or p.Ala444Pro mutant VPS13C does not accumulate at the cis- and medial-Golgi. **Fig. S7**: Wild type and p.Trp395Cys or p.Ala444Pro mutant VPS13C does not accumulate at the trans-Golgi. **Fig S8**: Western blot analysis of extracts from VPS13C knockout and wild type HeLa cells using the affinity-purified VPS13C antibody. **Fig. S9**: Haplotype sharing analysis. **Fig. S10**: *Trans *configuration of p.Met2711Ile/p.Ile2789Thr in patient P4 confirmed by allele specific PCR. **Fig. S11**: Family C. **Fig. S12**: *Cis* configuration of p.Met2764Ile/p.Val2765Leu in control C6. **Fig. S13**: Effect of c.4166-8C>A on *VPS13C *mRNA splicing.

## Data Availability

All data relevant to this study are included in the research paper or added to the supplementary file. The corresponding author will share additional information upon reasonable request. A previous preliminary version of this manuscript, that has not completed peer review, was present on the www.researchsquare.com repository as article/rs-29975. The current article is not published elsewhere.

## Abbreviations

AAI: age at inclusion
AAO: age at onset age
AD: Alzheimer’s disease
CADD: combined annotation dependent depletion
CCN6: cellular communication network factor 6
CLLU1: chronic lymphocytic leukemia up-regulated 1
DLB: dementia with Lewy bodies
ER: endoplasmic reticulum
FTD: frontotemporal dementia
LBD: Lewy body disease
LOF: loss of function
MAF: minor allele frequency
MoCA: montreal cognitive assessment
PD: Parkinson’s disease
PMD: post mortem delay
PTC: premature termination codon
RNF6: ring finger protein 6
SKAT-O: optimized sequence kernel association test
VPS13C: vacuolar protein sorting 13 homolog C
WGS: whole genome sequencing
WT: wild type
