# Supplementary material for: Contribution of rare homozygous and compound heterozygous VPS13C missense mutations to dementia with Lewy bodies and Parkinson’s disease
Source: Acta Neuropathol Commun. 2021 Feb 12;9:25. doi: 10.1186/s40478-021-01121-w (PMC7881566; doi:10.1186/s40478-021-01121-w)
Supplement: Supplementary file 1 — Additional file 1. Supplementary materials and methods. Detailed protocols and references. Supplementary results. Phasing compound heterozygous variants in the Belgian cohorts. Effect of VPS13C splice site variants on mRNA splicing. Supplementary tables. Table S1: Clinical and demographic characteristics of study cohorts. Table S2. Clinical data of patient carriers of rare homozygous or trans compound heterozygous VPS13C mutations. Table S3: Candidate genes and mutations identified in WGS data of family A. Table S4: VPS13C rare coding and splice site variants in DLB and PD patients and in controls. Table S5: Major genes associated with neurodegenerative brain diseases. Table S6: LBD patient carriers of cis compound heterozygous coding and splice site variants in VPS13C. Table S7: Control carriers of compound heterozygous coding and splice site variants in VPS13C. Table S8: Primer sequences. Table S9: In-silico predictions on VPS13C mRNA splicing of splice site variants in compound heterozygous carriers. Supplementary figures. Fig. S1: VPS13C rare coding and splice site variants in DLB (n=233) and PD patients (n=611), and in controls (844). Fig. S2: Late endosomal localization of VPS13C is lost in p.Trp395Cys and p.Ala444Pro mutations. Fig. S3: Lysosomal localization of VPS13C is lost in p.Trp395Cys and p.Ala444Pro mutations. Fig. S4: Missense mutations p.Trp395Cys and p.Ala444Pro disturb localization of VPS13C at the lysosomes. Fig. S5: Wild type and p.Trp395Cys or p.Ala444Pro mutant VPS13C does not accumulate at the endoplasmic reticulum. Fig. S6: Wild type and p.Trp395Cys or p.Ala444Pro mutant VPS13C does not accumulate at the cis- and medial-Golgi. Fig. S7: Wild type and p.Trp395Cys or p.Ala444Pro mutant VPS13C does not accumulate at the trans-Golgi. Fig S8: Western blot analysis of extracts from VPS13C knockout and wild type HeLa cells using the affinity-purified VPS13C antibody. Fig. S9: Haplotype sharing analysis. Fig. S10: Trans configuration of p.Met2711Ile/ [file 40478_2021_1121_MOESM1_ESM.docx]

**Contribution of rare homozygous and compound heterozygous *VPS13C* missense mutations to dementia with Lewy bodies and Parkinson’s disease**

Stefanie Smolders^1,2,3^, Stéphanie  Philtjens^1,2,3^, David Crosiers^1,2,3, 4^, Anne Sieben^1,2,6^, Elisabeth Hens^1,2,3,4,5^, Bavo Heeman^1,2,3^, Sara Van Mossevelde^1,2,4,5^, Philippe Pals^2,4^, Bob Asselbergh^1,2,3^, Roberto Dos Santos Dias^7^, Yannick Vermeiren^2,3^, Rik Vandenberghe^8^, Sebastiaan Engelborghs^2,3,9^, Peter Paul De Deyn^2,3,5^, Jean-Jacques Martin^2^, Patrick Cras^2,3,4^, Wim Annaert^7^, Christine Van Broeckhoven^1,2,3,#^, and BELNEU consortium

^1^Center for Molecular Neurology, VIB, Antwerp, Belgium

^2^Institute Born-Bunge, Antwerp, Belgium

^3^University of Antwerp, Antwerp, Belgium

^4^Department of Neurology, University Hospital Antwerp, Edegem, Belgium

^5^Department of Neurology, Hospital Network Antwerp, Antwerp, Belgium

^6^Department of Neurology, University Hospital Ghent and University of Ghent, Ghent, Belgium

^7^Center for Brain and Disease Research VIB, and Department of Neurosciences KU Leuven, Leuven, Belgium

^8^Department of Neurology, University Hospitals Leuven and Department of Neurosciences, KU Leuven, Leuven, Belgium

^9^Department of Neurology, UZ Brussel and Center for Neurosciences, Vrije Universiteit Brussel (VUB), Brussels, Belgium

^#^Corresponding author:

Prof. Dr. Christine Van Broeckhoven PhD DSc

Neurodegenerative Brain Diseases, VIB Center for Molecular Neurology

Department of Biomedical Sciences University of Antwerp

Universiteitsplein 1, 2610 Antwerp, Belgium

Tel: +32 3 265 1101, E-Mail: [Christine.VanBroeckhoven@uantwerpen.vib.be](mailto:Christine.VanBroeckhoven@uantwerpen.vib.be)

**SUPPLEMENTARY FILE**

**MATERIALS AND METHODS**

**Whole genome sequencing**

Short-read paired-end WGS of two siblings affected with DLB (family A, Fig. 1a), subsequent read alignment to the human reference genome (GRCh37/hg19) and base and variant calling were performed by Complete Genomics^TM^ Inc [4]. To annotate and select genetic variants in the WGS data we used GenomeComb [20]. High quality variants were selected based on a sequence coverage of at least 20X, a variant call score of ≥60 dB (Complete Genomics Inc.) and genomic location outside repeat regions marked as simple repeats or micro satellites by RepeatMasker v3.0 [25]. We selected coding and splice site variants with a potential impact on the protein sequence and a minor allele frequency (MAF) <1% in the 1000 Genome Project database [1] and below 25% in our in-house next generation sequencing database of Belgian patients with distinct neurological disorders. Priority was given to variants in line with autosomal recessive inheritance, i.e. homozygous or compound heterozygous variants.

**Resequencing of *VPS13C***

We performed PCR amplification of all 86 coding exons and flanking splice sites of *VPS13C* by amplicon-target PCR amplification (MASTR technology; Agilent). Amplicons were uniquely tagged based on the Nextera XT shotgun library preparation protocol (Illumina) [13]. Libraries (n=384) were pooled and sequenced in one run on the MiSeq platform using the MiSeq V3 chemistry (Illumina). After sample de-multiplexing, we mapped sequence reads using the Burrows-Wheeler Aligner (BWA) [16] to a mini-genome, combining the target sequences extracted from the human genome, reference sequence hg19. Sequence variants were called using GATKv3.5 HaplotypeCaller [3, 18] and variants annotated using GenomeComb [20]. Coding variants are numbered relative to the translation initiation codon in the largest *VPS13C* transcript (GenBank Accession Number NM_020821.2). Amino acid changes numbered according to the largest *VPS13C* isoform (GenPept Accession Number NP_065872.1). Sequencing reads, visualized with the Integrative Genomics Viewer (IGV) [21] using BAM files of individual samples.

**Sanger sequencing**

Primers were designed using the online Primer3 software [22]. Target regions were PCR amplified from genomic DNA and subsequently Sanger sequencing using the BigDye® Terminator Cycle Sequencing kit v3.1 (Applied Biosystems) on an ABI3730 automated sequencer (Applied Biosystems). Sanger sequences were analyzed using Seqman (DNASTAR) and NovoSNP software [26].

**Allele-specific PCR to determine allele-phase configuration**

Allele-specific PCR amplification was used to determine c*is*/*trans* configuration of two *VPS13C* missense mutations present in the same exon. For each mutation, an allele-specific and a wild-type primer were designed in combination with a general second primer using the online Primer3 software [22] to amplify both the wild-type and mutant allele separately (Table S8). The amplicon containing the location of the second mutation purified and Sanger sequenced (as described above).

**Haplotype sharing analysis**

For haplotype sharing, we selected 11 polymorphic STR markers surrounding *VPS13C* at chromosome 15q21 for genotyping: D15S1008, D15S198, D15S155, chr15:62080540-62080584, chr15:62440295-62440338, D15S1036, D15S997, D15S159, D15S993 and D15S1507. The STR markers were PCR amplified using fluorescently labelled primers and size-separated using GeneScan 500 Liz Size Standard (Applied Biosystems) on an ABI3730xl DNA Analyzer (Applied Biosystems). Local Genotype Viewer, used to score fragment lengths.

**Cell culture**

Lymphoblast cells, immortalized by Epstein Barr virus transformation of lymphocytes collected from whole blood on lithium heparin according to standard procedures [2, 7], were cultured in Roswell Park Memorial Institute 1640 medium (RPMI 1640; Life Technologies), supplemented with 15% fetal calf serum (Sigma Aldrich), 2 mM L-glutamine (Life Technologies) and 500 U/500 µg penicillin/streptomycin (Life Technologies).

Human cervical carcinoma (HeLa) cells were cultured in Modified Eagles medium (MEM; Life Technologies), supplemented with 10% fetal calf serum (Sigma Aldrich) and 500 U/500 µg penicillin/streptomycin (Life Technologies). Human neuroblastoma cells (SH-SY5Y) were cultured in Modified Eagles medium (MEM; Life Technologies) supplemented with 10% fetal calf serum (Sigma Aldrich), 1% non-essential amino acids (Life Technologies), 2 mM glutamine (Life Technologies) and 500 U/500 µg penicillin/streptomycin (Life Technologies).

**Long-read cDNA sequencing**

Total RNA was isolated and purified from lymphoblast cells using the RNeasy Mini Kit as recommended by the manufacturer (Qiagen), and subsequently treated with DNase (Turbo DNase Kit; Ambion). Polyadenylated RNA was enriched from total RNA samples using the Poly(A) RNA Selection Kit (Lexogen; M039100). First strand synthesis was performed on 50ng Poly A+ RNA by means of the SuperScript IV First-Strand Synthesis System using VN primer (VNP) and Template Switching Oligonucleotides (TSO) (Table S8), with both oligonucleotides containing sequence tags for subsequent second strand generation and PCR amplification using a Forward and Reverse primer (Table S8). cDNA amplification of *VPS13C* fragments containing the variants was performed using LongAmp® Taq DNA Polymerase (New England Biolabs) and primers designed with the online Primer3 software (Table S8) [22]. Native barcoding and adapter ligation were carried out using the Native Barcoding Expansion 1-12 (EXP-NBD104; Oxford Nanopore Technologies, ONT) in conjunction with the Ligation Sequencing Kit (SQK-LSK109; ONT). *VPS13C* amplicons from 11 individual samples were pooled equimolar and sequenced on a MinION, using a single FLO-MIN106 flow cell (ONT). In total 900Mb of data was generated (~650K reads). Base calling and barcode de-multiplexing of the raw data was performed with Guppy (v.3.2.2). Further analysis was performed with a pipeline integrated in GenomeComb [20]. Alignment to the hg38 reference sequence [24] was performed with minimap2 using the splicedhq preset [15]. Samtools was used for the conversion to BAM and sorting [17], which enabled visualization with IGV [21]. Single nucleotide variant calling and haplotyping was performed using longshot (v0.4.0) [5].

***In silico* prediction of splice-site variants**

For evaluation of the of splice site variants *in silico* we used five splicing prediction programs (SpliceSiteFinder-like, MaxEntScan, NNSPLICE and GeneSplicer) integrated in Alamut Visual version 2.11.0 (Interactive Biosoftware).

**Splice-site variant analysis on cDNA**

Lymphoblast cells were treated with 100 μg/ml cycloheximide (Sigma-Aldrich BVBA) or equal amounts of dimethyl sulfoxide (Fisher Scientific) for 4 h. The RNeasy procedure (RNeasy Mini Kit; Qiagen) was used to isolate and purify total RNA from lymphoblast cells as recommended by the manufacturer. Subsequently, total RNA was treated with DNase (Turbo DNase Kit; Ambion). cDNA synthesis of total RNA was performed primed with random hexamer primers using the SuperScript® III First-Strand Synthesis System for RT-PCR (Invitrogen), followed by a RNase H digestion to remove the RNA template from the cDNA:RNA hybrid molecule. To investigate the effect of *VPS13C* c.4166-8C>A on splicing, primers were designed using the online Primer3 software (Table S8) [22]. *VPS13C* cDNA was amplified in a total volume of 15 µl. Fragments were analyzed on a 2 % agarose gel, and sized using TrackIt™ 100 bp DNA Ladder (Invitrogen). Additionally, the *VPS13C* amplicons were purified and Sanger sequenced as described above.

**Quantitative RT-PCR**

Total RNA was isolated from lymphoblast cells, using the Ribopure kit (Ambion) or the Magtration Reagent MagDEA RNA 100 kit (Precision System Science), and was treated with DNase (Turbo DNase Kit; Ambion). First-strand cDNA was synthesized with the SuperScript III First-Strand Synthesis System (Invitrogen) utilizing random hexamer and oligo dT primers. PCR was carried out for 40 cycles with a *VPS13C*-specific forward and reverse primer (Table S8), using the Power SYBR Green PCR Master Mix (Life Technologies) on an ABI Viia™7 Real-Time PCR System (Applied Biosystems). Relative expression levels were quantified against two housekeeping genes, *TBP* and *hYWAZ*, using qbase software (Biogazelle, Ghent, Belgium).

**Generation of VPS13C antibody**

The cDNA encoding the amino acids 3621-3742 of human VPS13C, isoform 2A (NM_020821), was PCR amplified, using primers carrying the restriction sites for NheI and XhoI (Table S8), and subsequently cloned into a pET28a vector (Novagen), in frame with an N-terminal 6xHis tag (pET28-6xHis-VPS13C.2). The construct was transformed into BL21(DE3) competent E. coli (New England Biolabs) and expressed for 3h, at 28°C, to allow proper folding of the protein. Expressed recombinant protein was purified with Ni-NTA resin (Qiagen) followed by gel filtration chromatography using an ÄKTA™​ pure system equipped with a HiLoad 26/600 Superdex 75 pg column (GE Healthcare). Purity of recombinant protein was assessed by Coomassie Brilliant blue staining. New Zealand white rabbits were immunized with different amounts of purified recombinant protein, and the serum was subsequently tested by Western blot for VPS13C immunoreactivity, using VPS13C knockout HeLa cell extracts as a negative control (Fig. S8). Polyclonal antibodies from the sera displaying the highest titer were affinity purified against the recombinant protein immobilized on NHS-activated Sepharose® 4 Fast Flow (GE Healthcare) according to the manufacturer’s instructions. Antibodies were eluted from the column with 100mM Glycine pH 2.7 and immediately neutralized with 1M Tris-HCl pH 9.0. The eluted fraction was dialyzed against PBS for 48h, with three daily buffer changes. Finally, purified antibodies were concentrated to 1 mg/mL using a Vivaspin 15R, 30 000 MWCO hydrosart (Sartorius AG), and frozen in aliquots containing 50% glycerol.

**Western blotting**

Protein analysis performed in lymphoblast cells and brain lysates. Cells and brain tissue were lysed in modified radioimmune precipitation buffer (RIPA: 1% sodium dodecyl sulfate, 150 mM NaCl, 0.5% Na-Doc, 1% NP-40, 50 mM Tris-HCL; pH, 8.0) supplemented with protease and phosphatase inhibitor (2x Complete Protease inhibitor cocktail and 1x Phosphostop phosphatase inhibitor cocktail; both from Roche). Lysates were sonicated on ice, cleared at 20,000 g for 15 min at 4°C, and supernatants were collected for immunoblotting. Protein concentrations were measured using a BCA Protein Assay Kit (Pierce™; Thermo Fisher Scientific). Equal amounts of proteins were separated on a 3-8% Tris-Acetate gel (Life Technologies) and electro-blotted onto a polyvinylidene difluoride membrane (Hybond P; Amersham Biosciences). Membranes were blocked in 5% skimmed milk in PBS and probed with primary antibodies against VPS13C (polyclonal rabbit anti-human VPS13C (1:1,000; Novus Biologicals; NBP1-94043), polyclonal rabbit anti-human VPS13C (1:1,000; Novus Biologicals; NBP1-94044) or polyclonal rabbit anti-human (1:2,000; generated in house as described above)) and GAPDH (monoclonal mouse anti-human GAPDH (1:20,000; Genetex; GTX627408)). Immunodetection was performed with specific secondary antibodies conjugated to horse-radish peroxidase in combination with the ECL prime chemiluminescent detection system (GE Healthcare) or the WesternBright Sirius detection system (Isogen Life Science). The band intensities were determined by quantifying the mean pixel grey values in a rectangular region of interest using Image Quant TL software (GE Healthcare Life Sciences) and subsequently normalized to GAPDH.

**Preparation of cDNA constructs**

The codon optimized coding sequence of wild type (WT) human *VPS13C* was purchased in a Gateway® compatible pDONR vector from GeneArt^TM^ Gene synthesis (pDONR-VPS13C^WT^; Life Technologies). The *VPS13C* missense mutations p.Trp395Cys and p.Ala444Pro were introduced into the pDONR-VPS13C^WT^ vector with QuickChange Site-Directed Mutagenesis. Sequence verified mutant entry clones were subcloned into the Gateway® pcDNA™-DEST40 Vector with a C-terminal V5-6x His tag (Thermo Fisher Scientific) or into an in-house developed Gateway®-compatible pCR3 Vector with a C-terminal EmGFP-tag. Sequences were verified by DNA sequencing and primers used for cloning are listed in Table S8.

**Transfections**

HeLa or SH-SY5Y cells were transfected using either X-tremeGENE™ 9 DNA Transfection Reagents (Sigma Aldrich), Lipofectamine LTX plus (Invitrogen), both according to manufacturer’s protocol, or Polyethylenimine (PEI) according to an in house optimized protocol. Briefly, cells were seeded in a 6-well plate at 2.5x10^5^ cells per well, 24 hours before transfection. On the day of transfection, cell medium was replaced by medium without antibiotics. 1.44µg plasmid DNA was diluted in 115µl Opti-MEM (Life-Technologies) and in parallel, 7.21µl PEI was diluted 115µl Opti-MEM. The diluted PEI was added to the DNA and mixed gently by vortexing. After 10 min of incubation at room temperature, the solution was added to the cells. All cells were evaluated 48h post-transfection.

**Immunohistochemistry and live cell labeling**

HeLa or SH-SY5Y cells were grown on 12 mm glass coverslips (Fisher Scientific), fixed for 20 min with 4% paraformaldehyde (PFA; Laborimpex) in PBS at room temperature and washed three times in PBS. The cells were permeabilized in 0.25% Triton X-100 for 10 min, washed three times in PBS and blocked for 1h in 5% BSA (Merck) with normal donkey serum (1:500; Merck). Cells were then incubated overnight at 4°C with one or a combination of the following antibodies: monoclonal mouse anti-V5 tag (1:400; Life Technologies; R960-25), polyclonal goat anti-V5 tag (1:2,000; Abcam; ab9137), polyclonal rabbit anti-human Giantin (Golgi; 1:10,000; Covance; 924302), polyclonal rabbit anti-human TGN46 (Trans-Golgi Network; 1:800; Abcam; ab50595), monoclonal mouse anti-human PDI (endoplasmic reticulum (ER); 1:100; Abcam; ab2792), monoclonal mouse anti-human LAMP1 (lysosomal-associated membrane protein 1; 1:200; Developmental Studies Hybridoma Bank; H4A3), polyclonal rabbit anti-human Rab7 (late endosomes; 1:200; Sigma Aldrich; R4779) and monoclonal mouse anti-human CD63 (early endosomes; 1:250; Abcam, ab8219). To visualize the primary antibodies, the cells were incubated with secondary antibodies conjugated to Alexa Fluor 488, Alexa Fluor 594 or Alexa Fluor 647 (all 1:500; Thermo Fisher/Life Technologies) for 1h at room temperature.

For live cell imaging, transfected HeLa cells were plated on a standard 35 mm glass bottom dish (MatTek Corporation, P35G-1.5-14-C) and incubated for 30 min at 37°C with growth medium containing 50nM LysoTracker® probe (Thermo Fisher). After incubation, the medium was replaced by FluoroBrite DMEM (Life Technologies).

**Image acquisition and analysis**

Images were taken with a Zeiss LSM700 confocal microscope using either a 63x/1.40 Plan-Apochromat or a 40x/1.30 Plan-Neofluar objective. Filters, dichroics and scanning modes were set to exclude crosstalk between the different fluorescence channels, pixel sizes were set according to the Nyquist sampling theorem and z-stacks comprising entire cells, acquired at optimal step sizes. To visualize co-localization of VPS13C with different organelle markers, Fiji software generated line-intensity plot profiles [23]. For quantification of VPS13C mislocalization, two researchers who were blind for the genotype visually scored cells.

**RESULTS**

**Phasing compound heterozygous variants in the Belgian cohorts**

We identified one patient, P2, clinically diagnosed with DLB and homozygous for *VPS13C* p.Ala444Pro, located on two different haplotypes of which one haplotype was shared with the heterozygous p.Ala444Pro carriers in family A (Table 1, Fig. S9). Further, we observed 10 patients and 7 control individuals with rare compound heterozygous coding (with impact on protein sequence) and splice site variants in *VPS13C* (Table 1; Table S6; Table S7). We phased the compound heterozygous *VPS13C* variants to identify carriers with *trans* configuration.

The child of patient P3 (Family B) carried one *VPS13C* allele, p.Thr1218Ala, confirming *trans* configuration of p.Thr1218Ala/p.Ile2789Thr (Fig. 1a, Fig. S9). In patient P4, *trans* configuration of p.Met2711Ile/p.Ile2789Thr was confirmed by allele-specific PCR analysis (Fig. S10). Long-read cDNA sequencing of *VPS13C* transcript indicated *trans* configuration for p.Ala1687Val/p.Ser2904Leu in patient P5 and *cis* configuration for p.Thr933Ala/p.Leu3204Ser in patient P10. The two children of patient P11 in Family C were negative for both *VPS13C* missense mutations, p.Ser963Gly/p.Ser2026Phe, confirming *cis* configuration (Fig. S11). The p.Lys171Glu/c.4166-8C>A variants in patient P12 are located on the same haplotype, which was shared with three control individuals C3, C4 and C5, also carrying p.Lys171Glu/c.4166-8C>A in *cis* (Fig. S9). In control individual C6, p.Met2764Ile/p.Val2765Leu are located in *cis* based on their joint occurrence in sequencing reads obtained via targeted resequencing of *VPS13C* (Fig. S12). Long-read cDNA sequencing in control individual C7 identified *cis* configuration p.Ser2282Phe/p.Arg3176Gly.

We were unable to phase the compound heterozygous variants in patients P6, P7, P8 and P9 and controls C1 and C2 (Table 1; Table S7). DNA of relatives for segregation or biomaterials for RNA isolation were not available for the carriers P8, P9 and C1. Long-read ONT cDNA sequencing could not call the missense mutations in patients P6 and P7. In control individual C2, we were unable to amplify the *VPS13C* isoform 2, preventing phasing.

**Effect of VPS13C splice site variants on mRNA splicing**

Apart from the missense variants we also identified three splice site variants, c.448+7A>G, c.4056+3A>C and c.4166-8C>A, each partnering with a missense mutation, in carriers of compound heterozygous *VPS13C* variants (Table 1, Table S6, Table S7). The c.4056+3A>C variant in the PD patient P8 was absent in 664 controls and predicted to affect the canonical splice donor site of exon 36 by three *in silico* splicing prediction programs (Table S9). The splicing predictions for c.4166-8C>A and c.448+7A>G were inconsistent between prediction programs (Table S9). We did not observed exon skipping of c.4166-8C>A when treating the lymphoblast cells of the 4 carriers with cycloheximide before isolating total mRNA (Fig. S13). In control individual C2, we were unable to amplify the *VPS13C* isoform 2 specific exons 6 and 7, preventing evaluation of the effect of c.448+7A>G on cDNA derived of lymphoblast cells.

**TABLES AND FIGURES**

**Table S1:** **Clinical and demographic characteristics of study cohorts**

|  | **LBD patient cohort**  (PD + DLB patients) | **PD patient cohort** | **DLB patient cohort** | **Control cohort** |
| --- | --- | --- | --- | --- |
| **n** | 844 | 611 | 233 | 664 |
| **Gender, n (%)**  Females  Males | 267 (31.6)  577 (68.4) | 190 (31.1)  421 (68.9) | 77 (33.0)  156 (67.0) | 402 (60.5)  262 (39.5) |
| **AAO/AAI (years)**  Mean ± SD  Range | 62.9 ± 11.8  24-88 | 60.6 ± 11.3  24-88 | 70.2 ± 10.2  34-88 | 72.0 ± 9.4  34-88 |
| **Diagnosis, n (%)**  Clinical  Pathological | 770 (91.2)  74 (8.8) | 611 (100.0)  0 (0.0) | 159 (68.2)  74 (31.8) | -  - |
| **Positive familial history, n (%)**  Yes  No | 169 (20.0)  675 (80.0) | 113 (18.5)  498 (81.5) | 56 (24.0)  177 (76.0) | 0 (0.0)  664 (100.0) |

Note: The LBD cohort was genetically profiled for *GBA* using a custom-designed amplicon-target PCR amplification assay (MASTR technology, Agilent, Multiplicom, Niel, Belgium) and Sanger sequencing. Additionally, the PD cohort was genetically profiled for the 5 major PD genes (*SNCA*, *LRRK2*, *PARK2*, *PINK1* and *PARK7*) by means of Sanger sequencing for simple mutations and multiplex amplicon quantification (MAQ, Agilent, Multiplicom, Niel, Belgium), quantitative real-time PCR or multiplex ligation-dependent probe amplification (MLPA) [10] for copy number variants [19]. Abbreviations: LBD, Lewy body disease; PD, Parkinson’s disease; DLB, Dementia with Lewy bodies; AAO, age at onset; AAI, age at inclusion.

**Table S2. Clinical data of patient carriers of rare homozygous or *trans* compound heterozygous *VPS13C* mutations**

| **Patient** | **Diagnosis** | **Clinical features** | **Structural neuroimaging** | **Functional neuroimaging** | **CSF biomarkers** |
| --- | --- | --- | --- | --- | --- |
| P1 | DLB | Initially: deficiency of working memory, anxiety, fatigue, word finding difficulties, loss of initiative  Later: features of non-fluent aphasia, extrapyramidal signs of hypomimia, bradykinesia, propulsion, cog wheel rigidity and resting tremor, frontal disinhibition signs, myoclonus, social withdrawing, passivity, changed taste preferences, nightly hallucinations | MRI: slight bilateral prefrontal atrophy, later manifest temporal (L>R) and biparietal atrophy | SPECT: compatible with AD | Aβ_1-42_: 901.5pg/mL (Z-score 1.51)  Tau:14pg/mL (Z-score 1.66) |
| P2 | DLB | Bradykinesia, rigidity, tremor, postural instability, dementia (MMSE 17/30 at age 47), motor fluctuations, dyskinesia., Hoehn and Yahr stage 3, myoclonus. Neuropsychological testing: cortical (aphasia) and subcortical deficits (severe dysexecutive syndrome) | MRI: general atrophy, prominent left hemispheric atrophy | NA | NA |
| P3 | DLB | Initially: memory problems, difficulties in executive function, apathy, loss of initiative, bradypsychia, slightly broader base gait  Later: increased behavioral problems (nervosity, agression, perseveration), dysphasia evolving to akinetic mutism, myoclonia and later generalized tonic-clonic epileptic seizures, extrapyramidal signs of hypokinesia, propulsion | NA | SPECT: slight hypoperfusion in frontal regions | NA |
| P4 | DLB | Gait instability, fluctuating cognitive difficulties, disorientation in time, episodic confusions, visual hallucinations, word retrieval difficulties, occasional nightly wandering behavior; extrapyramidal signs of hypomimia, propulsia, small shuffling gait, decreased arm swing, disturbed postural reflexes, cog wheel rigidity, intermittent resting, postural and intention tremor, hypokinesia, slight dysartria, dysmetria of upper limbs | MRI: frontotemporal atrophy | NA | NA |

**Table S2. Clinical data of patient carriers of rare homozygous or *trans* compound heterozygous *VPS13C* mutations (continued)**

| **Patient** | **Diagnosis** | **Clinical features** | **Structural neuroimaging** | **Functional neuroimaging** | **CSF biomarkers** |
| --- | --- | --- | --- | --- | --- |
| P5 | PD | Bradykinesia, rigidity, depression, MMSE 29/30 at age 60, no motor fluctuations, no dyskinesia, Hoehn and Yahr stage 1.5, UPDRS motor score 16 | NA | NA | NA |
| P6 | DLB | Bradykinesia, tremor, dementia (MMSE 17/30 at age 75). Neuropsychological testing: cortical (severe deficits of memory, attention, problem solving, severe dysexecutive syndrome) and subcortical deficits (hypophone dysartry, deficits in initiation, perseveration, closing in phenomenon) | MRI: subcortical atrophy, mild chronic microvascular lesions | DATscan abnormal SPECT: severe relative hypoperfusion frontoparietal (L>R), relative hypoperfusion frontal (L>R), discrete relative hypoperfusion cerebellar (R), chronic ischaemic brain lesions | NA |
| P7 | PD | Bradykinesia, rigidity, tremor, postural instability, no motor fluctuations, no dyskinesia., Hoehn and Yahr stage 1 | NA | NA | NA |
| P8 | PD | Bradykinesia, rigidity, no motor fluctuations, no dyskinesia, Hoehn and Yahr stage 2 | NA | DATscan: abnormal | NA |
| P9 | PD | Bradykinesia, rigidity, tremor | NA | NA | NA |

Note: The Hoehn and Yahr scale is a commonly used system for describing the progression of Parkinson's disease symptoms [9]. Abbreviations: MCI, mild cognitive impairment; AD, Alzheimer’s disease; CSF, cerebrospinal fluid; MRI, magnetic resonance imaging; NA, not available; R, right; L, left; SPECT, single photon emission computed tomography; CT, computerized tomography; EEG, electroencephalogram; DATscan, dopamine transporter imaging; MMSE, Mini-Mental State Examination [6]; UPDRS, Unified Parkinson's Disease Rating Scale [8].

**Table S3. Candidate genes and mutations identified in WGS data of family A**

| **Gene** | **∆CDS^a^** | **∆AA^b^** | **MAF gnomAD_nfe (%)** | **CADD_Phred score^c^** |
| --- | --- | --- | --- | --- |
| *CCN6* | c.131C>T | p.P44L^#^ | 0.274 | 0.991 |
|  | c.145C>A | p.P49T^#^ | 0.273 | 5.708 |
| *CLLU1* | c.123insA | p.N41Kfs*3^#^ | 0.851 | 17.25 |
|  | c.217A>G | p.I73V^#^ | 0.845 | 4.759 |
|  | c.317C>T | p.T106M^#^ | 0.883 | 1.791 |
| *RNF6* | c.568A>G | p.T190A^#^ | 0.234 | 1.646 |
|  | c.1664C>A | p.T555N^#^ | 0.0101 | 9.472 |
| *VPS13C* | c.1185G>C | p.W395C^§^ | - | 33 |
|  | c.1330G>C | p.A444P^§^ | 0.00864 | 28.4 |

Note: ^a^Coding nomenclature according to NM_198239 (*CCN6*), NM_001025233 (*CLLU1*), NM_183044 (*RNF6*) and NM_020821 (*VPS13C*); ^b^Protein nomenclature according to NP_937882 (CCN6), NP_001020404 (CLLU1), NP_898865 (RNF6) and NP_0658721 (*VPS13C*); ^c^CADD_Phred, Combined Annotation Dependent Depletion prediction score [11]; ^#^Mutations in *cis* configuration; ^§^Mutations in *trans* configuration. Abbreviations: ∆CDS, coding sequence substitution; ΔAA, amino acid substitution; MAF, minor allele frequency; gnomAD_nfe, Genome Aggregation Database non-Finnish European population [14].

**Table S4. *VPS13C* rare coding and splice site variants in DLB and PD patients and in controls**

| **∆CDS^a^** | **ΔAA^b^** | **MAF gnomAD_nfe (%)** | **CADD_Phred score^c^** | **MAF DLB**  **patients (%) n=233** | **MAF PD**  **patients (%) n=611** | **MAF control individuals (%) n=664** | **MAF PD, DLB and control cohort (%) n=1506** |
| --- | --- | --- | --- | --- | --- | --- | --- |
| c.11245C>T | p.P3749S | - | 11.63 | 0.0000 | 0.0818 | 0.0000 | 0.0332 |
| c.11176A>G | p.I3726V | 0.594 | 23.1 | 0.2146 | 0.2455 | 0.9036 | 0.564 |
| c.11076+3A>G | - | 0.003993 | 17 | 0.2146 | 0.0000 | 0.0000 | 0.0332 |
| c.10955G>A | p.R3652Q | 0.0605 | 29.4 | 0.0000 | 0.0818 | 0.0000 | 0.0332 |
| c.10954C>T | p.R3652* | 0.0283 | 49 | 0.0000 | 0.0000 | 0.0753 | 0.0332 |
| c.10850C>G | p.S3617C | 0.00176 | 10.19 | 0.0000 | 0.0000 | 0.0753 | 0.0332 |
| c.10784G>A | p.R3595H | 0.00155 | 35 | 0.0000 | 0.0000 | 0.0753 | 0.0332 |
| c.10600G>A | p.G3534R | 0 | 34 | 0.0000 | 0.0818 | 0.0000 | 0.0332 |
| c.10239G>A | p.M3413I | 0.00165 | 26.1 | 0.0000 | 0.0818 | 0.0000 | 0.0332 |
| c.10237A>T | p.M3413L | - | 22.7 | 0.0000 | 0.0000 | 0.0753 | 0.0332 |
| c.9703T>G | p.S3235A | 0.000892 | 24 | 0.0000 | 0.0818 | 0.0000 | 0.0332 |
| c.9611T>C | p.L3204S | 0.0158 | 31 | 0.2146 | 0.0000 | 0.0000 | 0.0332 |
| c.9564G>T | p.Q3188H | 0.000892 | 20.5 | 0.0000 | 0.0818 | 0.0000 | 0.0332 |
| c.9526C>G | p.R3176G | 0.230 | 17.53 | 0.2146 | 0.2455 | 0.6777 | 0.431 |
| c.9470A>C | p.D3157A | - | 24.2 | 0.0000 | 0.0000 | 0.0753 | 0.0332 |
| c.8845A>G | p.N2949D | - | 10.55 | 0.0000 | 0.0818 | 0.0000 | 0.0332 |
| c.8844+6T>A | - | - | 11.76 | 0.0000 | 0.0818 | 0.0000 | 0.0332 |
| c.8711C>T | p.S2904L | 0.343 | 26.7 | 0.0000 | 0.2455 | 0.3765 | 0.265 |
| c.8668G>C | p.D2890H | 0.00854 | 22.4 | 0.0000 | 0.1637 | 0.0753 | 0.0995 |
| c.8423A>G | p.K2808R | 0.0477 | 21.8 | 0.2146 | 0.0818 | 0.0753 | 0.0995 |
| c.8366T>C | p.I2789T | 1.00 | 2.797 | 0.8584 | 0.4092 | 0.6777 | 0.597 |
| c.8293G>C | p.V2765L | 0.000879 | 20.4 | 0.0000 | 0.0000 | 0.0753 | 0.0332 |
| c.8292G>T | p.M2764I | - | 19.55 | 0.0000 | 0.0000 | 0.0753 | 0.0332 |
| c.8133G>A | p.M2711I | 0.00177 | 22.5 | 0.2146 | 0.0000 | 0.0753 | 0.0663 |
| c.8071C>G | p.H2691D | 0.0112 | 20.7 | 0.0000 | 0.0818 | 0.0000 | 0.0332 |
| c.7688A>G | p.N2563S | 0.0230 | 5.788 | 0.2146 | 0.1637 | 0.0753 | 0.133 |
| c.7644A>C | p.K2548N | - | 25 | 0.0000 | 0.0000 | 0.0753 | 0.0332 |
| c.7628C>G | p.S2543C | - | 28.9 | 0.0000 | 0.0818 | 0.0000 | 0.0332 |
| c.7618A>G | p.T2540A | 0.00360 | 24.6 | 0.0000 | 0.0818 | 0.0753 | 0.0663 |
| c.7276G>A | p.V2426I | 0.244 | 12.31 | 0.0000 | 0.1637 | 0.2259 | 0.166 |
| c.7169C>T | p.T2390I | - | 31 | 0.0000 | 0.0000 | 0.0753 | 0.0332 |
| c.7139A>G | p.H2380R | 0.4156 | 9.482 | 0.6438 | 0.6547 | 0.3765 | 0.531 |

**Table S4. *VPS13C* rare coding and splice site variants in DLB and PD patients and in controls (continued)**

| **∆CDS^a^** | **ΔAA^b^** | **MAF gnomAD_nfe (%)** | **CADD_Phred score^c^** | **MAF DLB**  **patients (%) n=233** | **MAF PD**  **patients (%) n=611** | **MAF control individuals (%) n=664** | **MAF PD, DLB and control cohort (%) n=1506** |
| --- | --- | --- | --- | --- | --- | --- | --- |
| c.7043A>G | p.Q2348R | 0 | 0.017 | 0.0000 | 0.0818 | 0.0000 | 0.0332 |
| c.6995T>A | p.I2332N | 0.00310 | 12.75 | 0.0000 | 0.0000 | 0.0753 | 0.0332 |
| c.6845C>T | p.S2282F | 0.0178 | 24.8 | 0.0000 | 0.0000 | 0.0753 | 0.0332 |
| c.6821A>G | p.E2274G | 0.00176 | 26.7 | 0.0000 | 0.0000 | 0.0753 | 0.0332 |
| c.6815T>C | p.I2272T | 0.107 | 0.009 | 0.0000 | 0.1637 | 0.0753 | 0.0995 |
| c.6723C>G | p.I2241M | - | 8.514 | 0.0000 | 0.0000 | 0.0753 | 0.0332 |
| c.6077C>T | p.S2026F | 0.00354 | 21.7 | 0.0000 | 0.0818 | 0.0000 | 0.0332 |
| c.5771C>T | p.T1924I | 0.000955 | 10.99 | 0.2146 | 0.0000 | 0.0000 | 0.0332 |
| c.5537T>C | p.L1846S | - | 26.8 | 0.0000 | 0.0818 | 0.0000 | 0.0332 |
| c.5410A>G | p.I1804V | 0.000777 | 3.377 | 0.0000 | 0.0000 | 0.0753 | 0.0332 |
| c.5146T>G | p.L1716V | - | 21.4 | 0.0000 | 0.0818 | 0.0000 | 0.0332 |
| c.5060C>T | p.A1687V | 0.000881 | 22.5 | 0.0000 | 0.0818 | 0.1506 | 0.0995 |
| c.4166-8C>A | - | 0.159 | 11.58 | 0.0000 | 0.2455 | 0.2259 | 0.199 |
| c.4129G>A | p.D1377N | - | 15.61 | 0.0000 | 0.0818 | 0.0000 | 0.0332 |
| c.4056+3A>C | - | 0.00705 | 14.06 | 0.0000 | 0.0818 | 0.0000 | 0.0332 |
| c.3713C>G | p.R1238H | 0.00851 | 35 | 0.0000 | 0.0000 | 0.0753 | 0.0332 |
| c.3652A>G | p.T1218A | 0.000781 | 26.4 | 0.2146 | 0.0000 | 0.0000 | 0.0332 |
| c.3613A>T | p.N1205Y | 0.00238 | 26.3 | 0.0000 | 0.0000 | 0.0753 | 0.0332 |
| c.3560G>C | p.C1187S | 0.000889 | 24.2 | 0.0000 | 0.0000 | 0.2259 | 0.0995 |
| c.3515C>T | p.T1172I | 0.00707 | 15.29 | 0.0000 | 0.0000 | 0.0753 | 0.0332 |
| c.2887A>G | p.S963G | 0.00541 | 16.17 | 0.0000 | 0.0818 | 0.0000 | 0.0332 |
| c.2797A>G | p.T933A | 0.577 | 12.67 | 0.4292 | 0.8183 | 0.4518 | 0.597 |
| c.2618-1G>A | - | - | 33 | 0.2146 | 0.0000 | 0.0000 | 0.0332 |
| c.2485A>G | p.M829V | 0.01166 | 0.944 | 0.0000 | 0.0000 | 0.0753 | 0.0332 |
| c.2296A>G | p.T766A | - | 12.18 | 0.0000 | 0.0818 | 0.0753 | 0.0663 |
| c.2216A>G | p.E739G | - | 27.4 | 0.0000 | 0.0000 | 0.0753 | 0.0332 |
| c.1880T>C | p.V627A | 0.00698 | 19.83 | 0.0000 | 0.0000 | 0.0753 | 0.0332 |
| c.1693T>A | p.S565T | 0.0396 | 8.512 | 0.0000 | 0.0818 | 0.0753 | 0.0663 |
| c.1538G>A | p.G513D | 0.00265 | 32 | 0.2146 | 0.0000 | 0.0000 | 0.0332 |
| c.1330G>C | p.A444P | 0.00864 | 28.4 | 0.6438 | 0.3273 | 0.0000 | 0.232 |
| c.1302G>A | p.K434N | - | 23.1 | 0.0000 | 0.0000 | 0.0753 | 0.0332 |
| c.1185G>C | p.W395C | - | 33 | 0.2146 | 0.0000 | 0.0000 | 0.0332 |

**Table S4. *VPS13C* rare coding and splice site variants in DLB and PD patients and in controls (continued)**

| **∆CDS^a^** | **ΔAA^b^** | **MAF gnomAD_nfe (%)** | **CADD_Phred score^c^** | **MAF DLB**  **patients (%) n=233** | **MAF PD**  **patients (%) n=611** | **MAF control individuals (%) n=664** | **MAF PD, DLB and control cohort (%) n=1506** |
| --- | --- | --- | --- | --- | --- | --- | --- |
| c.970A>G | p.I324V | 0.00620 | 0.01 | 0.0000 | 0.0818 | 0.0000 | 0.0332 |
| c.950C>T | p.T317M | 0.00776 | 28.9 | 0.0000 | 0.1637 | 0.0000 | 0.0663 |
| c.917A>G | p.Y306C | 0.00698 | 12.14 | 0.0000 | 0.0000 | 0.0753 | 0.0332 |
| c.864A>G | p.I288M | 0.00314 | 8.875 | 0.0000 | 0.0000 | 0.0753 | 0.0332 |
| c.716T>C | p.L239S | 0.00312 | 27.7 | 0.0000 | 0.0818 | 0.0000 | 0.0332 |
| c.511A>G | p.K171E | 0.118 | 11.79 | 0.0000 | 0.0818 | 0.2259 | 0.133 |
| c.448+7A>G | - | 0.00932 | 15.69 | 0.0000 | 0.0000 | 0.0753 | 0.0332 |

Note: Targeted resequencing of *VPS13C* identified 71 or splice site variants with a potential impact on the protein sequence and with a MAF ≤ 1%. ^a^Coding nomenclature according to NM_020821; ^b^Protein nomenclature according to NP_0658721; ^c^CADD_Phred, Combined Annotation Dependent Depletion prediction score [11]. Abbreviations: ∆CDS, coding sequence substitution; ΔAA, amino acid substitution; MAF, minor allele frequency; gnomAD_nfe, Genome Aggregation Database non-Finnish European population [14].

**
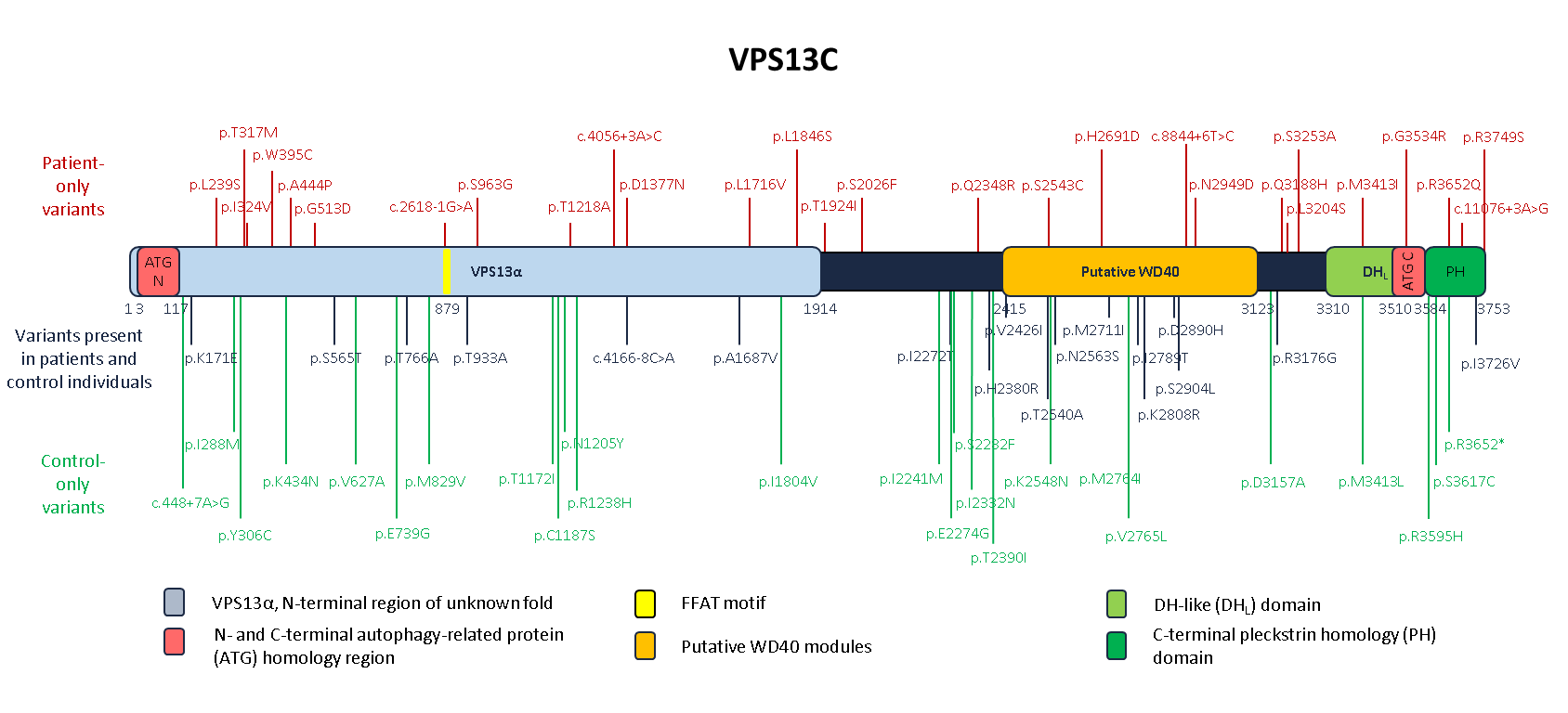
**

**Fig. S1. *VPS13C* rare coding and splice site variants in DLB (n=233) and PD patients (n=611), and in controls (844).** Linear representation of all genetic variants in *VPS13C* (n=71) identified*.* Domains are based on [12], protein nomenclature according to NP_0658721. Variants in red (n=28) are present only in patients, variants in blue (n= 18) are in patients and control individuals and variants in green (n=25) are only in in control individuals.

**Table S5. Major genes associated with neurodegenerative brain diseases.**

| **PD** | **AD** | **FTD** | **ALS** | **Prion disease** |
| --- | --- | --- | --- | --- |
| *LRRK2*  *SNCA*  *PARK2*  *PINK1*  *PARK7*  *VPS13C*  *VPS35*  *EIF4G1*  *FBXO7*  *ATP13A2*  *GBA*  *AMBRA1* | *APP*  *PSEN1*  *PSEN2*  *APOE*  *CLU*  *CR1*  *PICALM*  *BIN1*  *CSF1R* | *GRN*  *C9orf72*  *MAPT*  *VCP*  *CHMP2B* | *TARDBP*  *FUS*  *SOD1*  *ELP3* | *PRNP* |

Note: Whole exome sequencing of DLB carriers was performed to exclude known pathogenic mutations. Abbreviations: PD, Parkinson’s disease; AD, Alzheimer’s disease; FTD, frontotemporal dementia; ALS, amyotrophic lateral sclerosis.

**Table S6. LBD patient carriers of *cis* compound heterozygous coding and splice site variants in *VPS13C***

| **Patient** | **Dx** | **AAO** | **∆CDS^a^** | **ΔAA^b^** | **MAF gnomAD_nfe (%)** | **CADD_**  **Phred score^c^** | **MAF patient cohort (%) n=844** | **MAF control cohort (%) n=664** | **Phase** | **F in patients (%)** | **F in**  **controls**  **(%)** | **F expected^d^ (%)** |
| --- | --- | --- | --- | --- | --- | --- | --- | --- | --- | --- | --- | --- |
| P10* | DLB | 61 | c.2797A>G | p.Thr933Ala | 0.577 | 12.67 | 0.711 | 0.452 | *Cis* | 0.118 | 0 | 0.000198 |
|  |  |  | c.9611T>C | p.Leu3204Ser | 0.0158 | 31 | 0.0592 | 0 |  |  |  |  |
| P11* | PD | 67 | c.2887A>G | p.Ser963Gly | 0.00541 | 16.17 | 0.0592 | 0 | *Cis* | 0.118 | 0 | 0.0000110 |
|  |  |  | c.6077C>T | p.Ser2026Phe | 0.00354 | 21.7 | 0.0592 | 0 |  |  |  |  |
| P12* | PD | 70 | c.511A>G | p.Lys171Glu | 0.118 | 11.79 | 0.0592 | 0.226 | *Cis* | 0.118 | 0.452 | 0.000265 |
|  |  |  | c.4166-8C>A | - | 0.159 | 11.58 | 0.178 | 0.226 |  |  |  |  |

*Lymphoblast cells available. ^a^Coding nomenclature according to NM_020821; ^b^Protein nomenclature according to NP_0658721; ^c^CADD_Phred, score [11]. ^d^ Expected frequency is calculated according to the Hardy–Weinberg principle, using the MAF of the single alleles in patients plus controls (n=1508); Abbreviations: Dx diagnosis; PD, Parkinson’s disease; DLB, Dementia with Lewy bodies; ∆CDS coding sequence substitution; ΔAA amino acid substitution, MAF, minor allele frequency, F frequency of homozygotes and compound heterozygotes; AAO, age at onset; gnomAD_nfe, Genome Aggregation Database non-Finnish European population [14].

**Table S7. Control carriers of compound heterozygous coding and splice site variants in *VPS13C***

| **Control** | **AAI** | **∆CDS^a^** | **ΔAA^b^** | **MAF gnomAD_nfe (%)** | **CADD_Phred score^c^** | **MAF patient cohort (%) n=844** | **MAF control cohort (%) n=664** | **Phase** | **F in patients (%)** | **F in**  **controls**  **(%)** | **F expected^d^ (%)** |
| --- | --- | --- | --- | --- | --- | --- | --- | --- | --- | --- | --- |
| C1 | 59 | c.3515C>T | p.Thr1172Ile | 0.00707 | 15.29 | 0 | 0.0754 | Unknown | 0 | 0.151 | 0.000143 |
|  |  | c.9526C>G | p.Arg3176Gly | 0.230 | 17.53 | 0.237 | 0.679 |  |  |  |  |
| C2* | 68 | c.448+7A>G | - | 0.00932 | 15.69 | 0 | 0.0754 | Unknown | 0 | 0.151 | 0.0000330 |
|  |  | c.5060C>T | p.Ala1687Val | 0.314 | 22.5 | 0.0592 | 0.151 |  |  |  |  |
| C3* | 81 | c.511A>G | p.Lys171Glu | 0.118 | 11.79 | 0.0592 | 0.226 | *Cis* | 0.118 | 0.452 | 0.000265 |
|  |  | c.4166-8C>A | - | 0.159 | 11.58 | 0.178 | 0.226 |  |  |  |  |
| C4* | 78 | c.511A>G | p.Lys171Glu | 0.118 | 11.79 | 0.0592 | 0.226 | *Cis* | 0.118 | 0.452 | 0.000265 |
|  |  | c.4166-8C>A | - | 0.159 | 11.58 | 0.178 | 0.226 |  |  |  |  |
| C5* | 77 | c.511A>G | p.Lys171Glu | 0.118 | 11.79 | 0.0592 | 0.226 | *Cis* | 0.118 | 0.452 | 0.000265 |
|  |  | c.4166-8C>A | - | 0.159 | 11.58 | 0.178 | 0.226 |  |  |  |  |
| C6 | 64 | c.8292G>T | p.Met2764Ile | - | 19.55 | 0 | 0.0754 | *Cis* | 0 | 0.151 | 0.0000110 |
|  |  | c.8293G>C | p.Val2765Leu | 0.000879 | 20.4 | 0 | 0.0754 |  |  |  |  |
| C7* | 62 | c.6845C>T | p.Ser2282Phe | 0.0178 | 24.8 | 0 | 0.0754 | *Cis* | 0 | 0.151 | 0.000143 |
|  |  | c.9526C>G | p.Arg3176Gly | 0.230 | 17.53 | 0.237 | 0.679 |  |  |  |  |

Note: Control individuals were not assessed for motor symptoms. *Carriers with lymphoblast cells available. ^a^Coding nomenclature according to NM_020821; ^b^Protein nomenclature according to NP_0658721; ^c^CADD_Phred, Combined Annotation Dependent Depletion prediction score [11]; ^d^Frequency calculated according to the Hardy–Weinberg principle, using the MAF of single alleles in the PD, DLB and control cohort (n=1508). Abbreviations: ∆CDS, coding sequence substitution; ΔAA, amino acid substitution; MAF, minor allele frequency; F, frequency of compound heterozygotes or homozygotes; AAI, age at inclusion; gnomAD_nfe, Genome Aggregation Database non-Finnish European population [14].


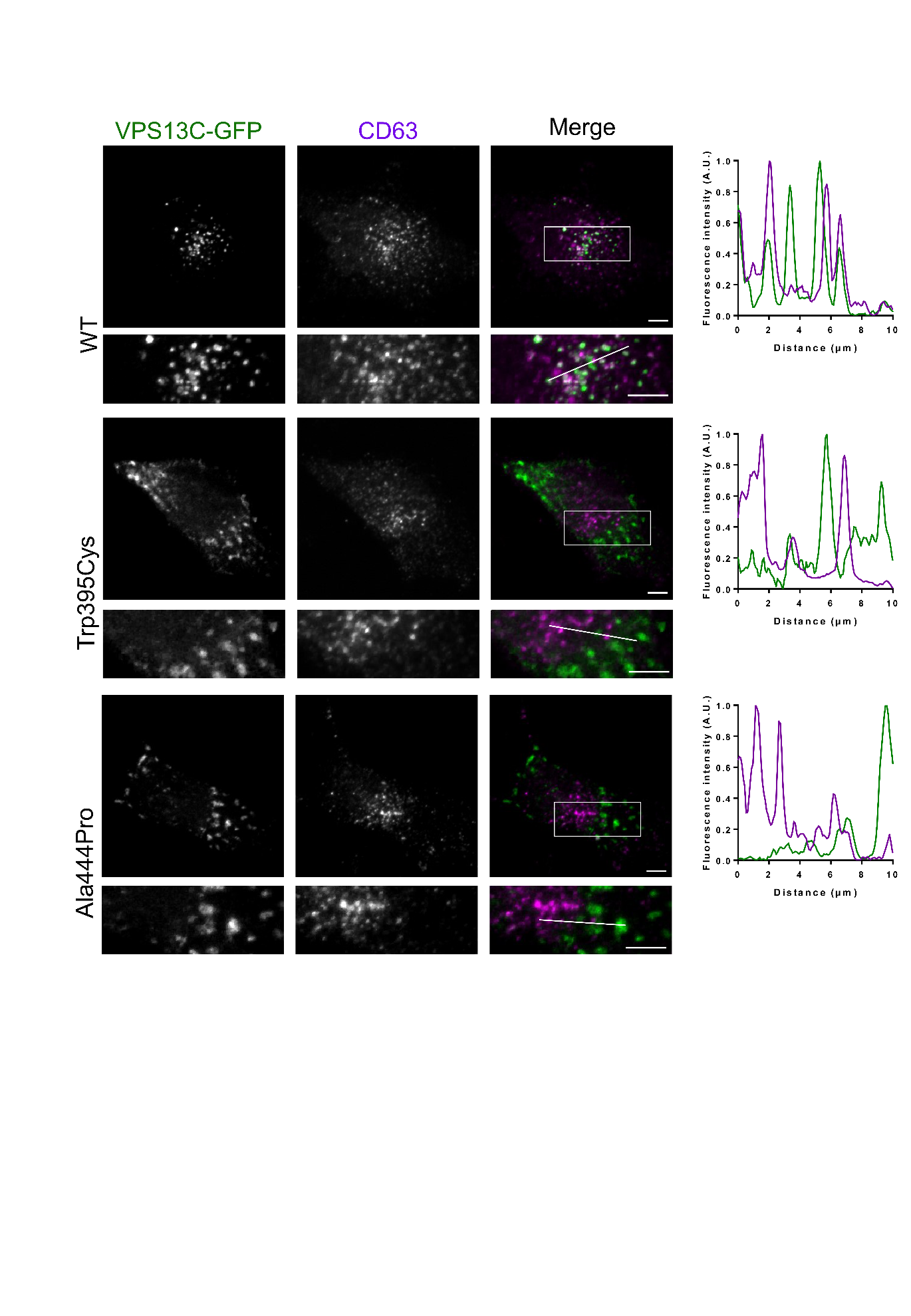


**Fig. S2. Late endosomal localization of VPS13C is lost in p.Trp395Cys and p.Ala444Pro mutations.** HeLa cells were transfected with either wild type or mutant (p.Trp395Cys or p.Ala444Pro) VPS13C construct containing a C-terminal GFP-tag. GFP fluorescence was used to visualize VPS13C (green) and an immune-fluorescent staining against CD63 (magenta) was used to stain late endosomes. Cells expressing wild type VPS13C showed a vesicular staining pattern that co-localized with late endosomes, while mutant VPS13C accumulates at larger cytosolic structures that do not co-localize with late endosomes. White lines in the zoom indicate the site used for the intensity line plots. White square = zoom. Scale bar = 5µm.


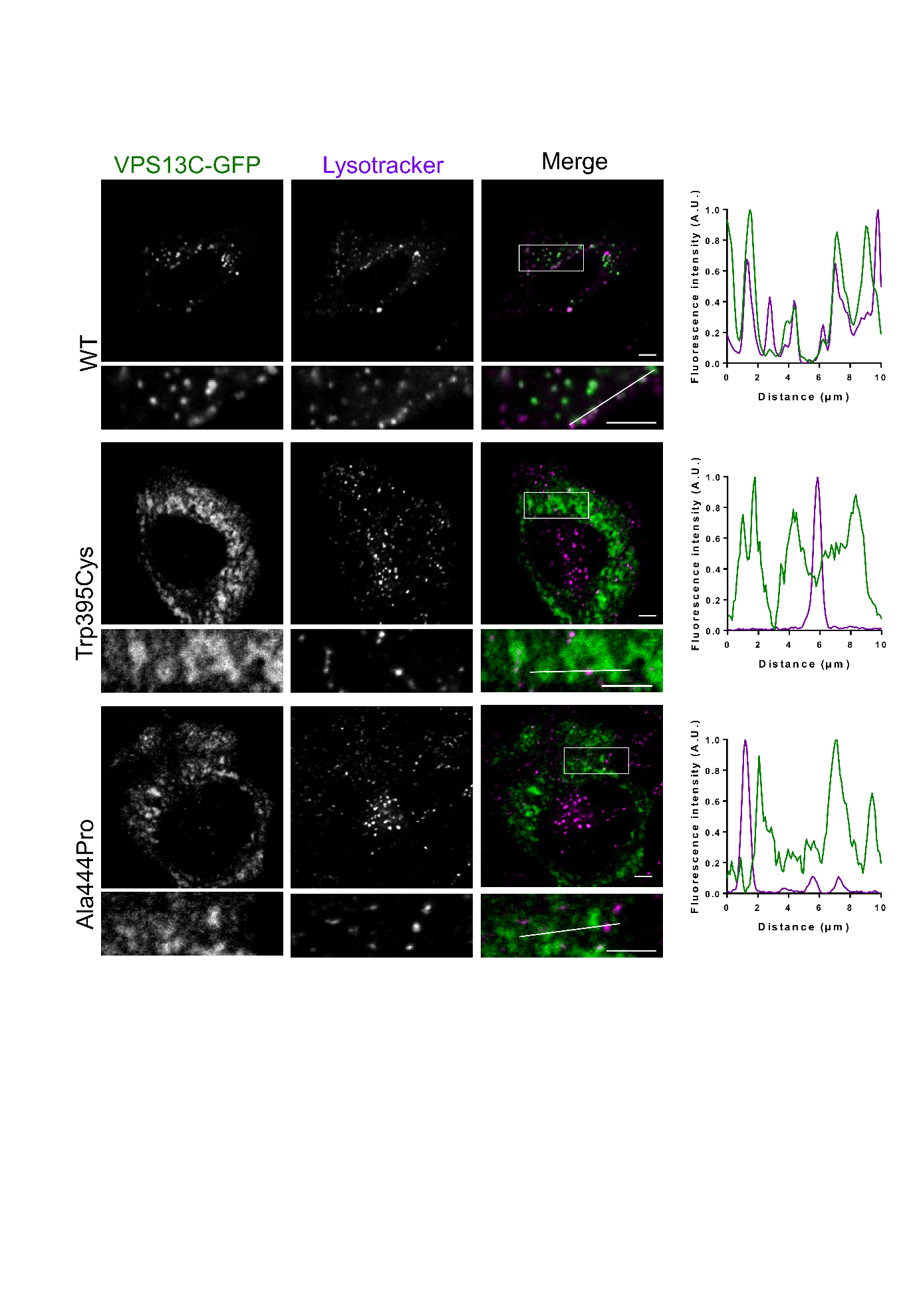


**Fig. S3. Lysosomal localization of VPS13C is lost in p.Trp395Cys and p.Ala444Pro mutations.** HeLa cells were transfected with either wild type or mutant (p.Trp395Cys or p.Ala44Pro) VPS13C construct containing a C-terminal GFP-tag. The GFP fluorescence was used to visualize VPS13C (green) and lysotracker Red (magenta) was used to stain acidic vesicles, such as lysosomes. Cells expressing wild type VPS13C showed a vesicular staining pattern that co-localized with lysosomes. Intensity line plot shows the overlap between wild type VPS13C and lysosomes. Mutant VPS13C accumulates in larger cytosolic structures that do not co-localize with lysosomes. White lines in the zoom indicate the site used for the intensity line plots. White square = zoom. Scale bar = 5µm.


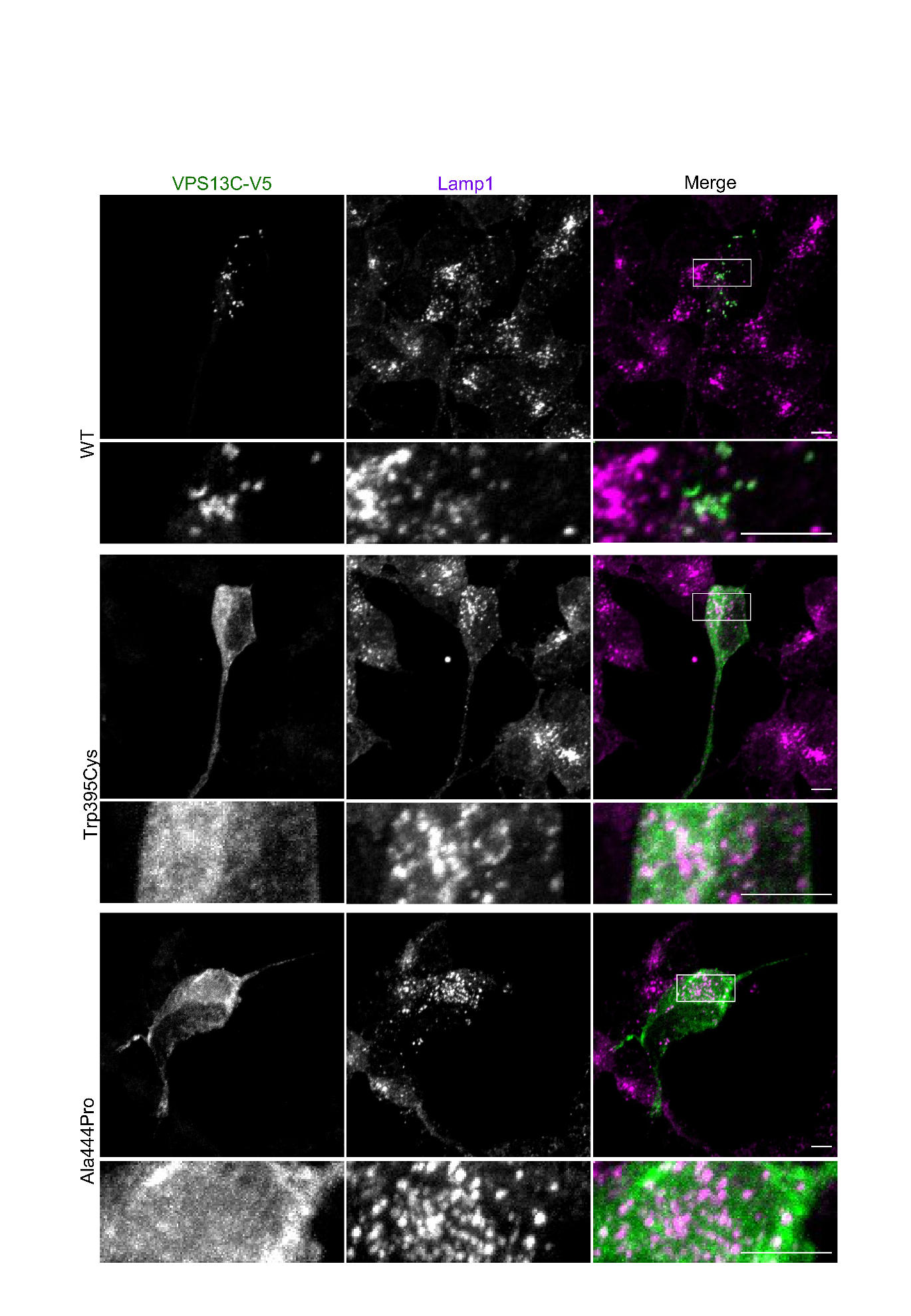


**Fig. S4. Missense mutations p.Trp395Cys and p.Ala444Pro disturb localization of VPS13C at the lysosomes.** SH-SY5Y cells were transfected with either wild type or mutant (p.Trp395Cys or p.Ala444Pro) VPS13C construct containing a C-terminal GFP-tag. GFP fluorescence was used to visualize VPS13C (green) and an immune-fluorescent staining against Lamp1 (magenta) was used to stain the lysosomes. Cells expressing VPS13C wild type showed a clear vesicular staining pattern while those expressing the missense mutations p.Trp395Cys or p.Ala444Pro mislocalized in larger cytosolic structures. White square = zoom. Scale bar = 5µm.


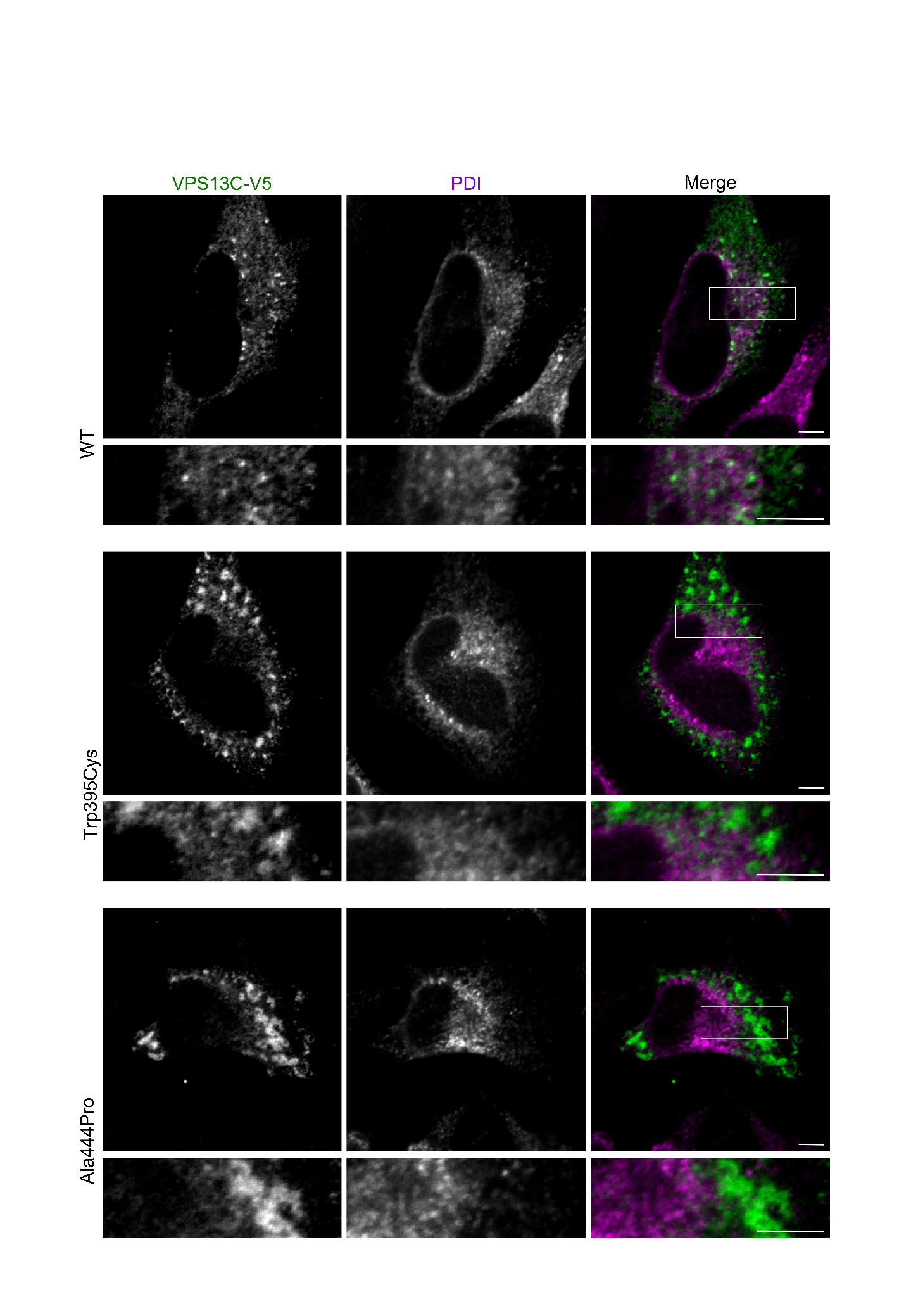


**Fig. S5. Wild type and p.Trp395Cys or p.Ala444Pro mutant VPS13C does not accumulate at the endoplasmic reticulum.** HeLa cells were transfected with either wild type or mutant (p.Trp395Cys or p.Ala444Pro) VPS13C construct containing a C-terminal V5-tag. HeLa cells were double stained with an anti-V5-tag antibody to visualize VPS13C (green) and an antibody staining the endoplasmic reticulum (PDI, magenta). No co-localization of VPS13C with the endoplasmic reticulum could be observed for wild type or mutated VPS13C. White square = zoom. Scale bar = 5µm.


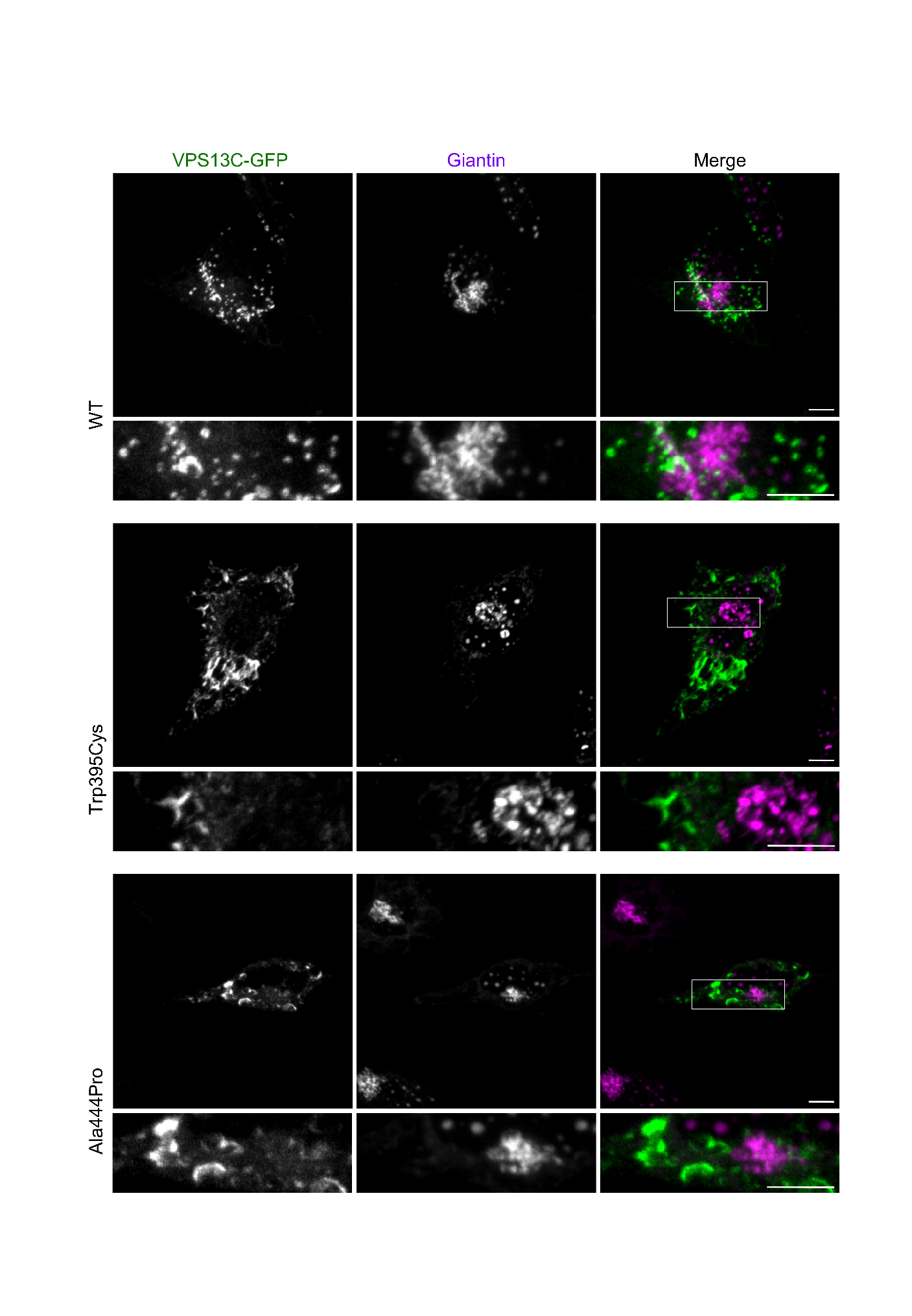


**Fig. S6. Wild type and p.Trp395Cys or p.Ala444Pro mutant VPS13C does not accumulate at the cis- and medial-Golgi.** HeLa cells were transfected with either wild type or mutant (p.Trp395Cys or p.Ala444Pro) VPS13C construct containing a C-terminal GFP-tag. GFP fluorescence was used to visualize VPS13C (green) and an immune-fluorescent staining against Giantin (magenta) was used to stain the cis- and medial-Golgi. No co-staining of VPS13C with the cis- and medial Golgi could be observed for wild type or mutated VPS13C. White square = zoom. Scale bar = 5µm.


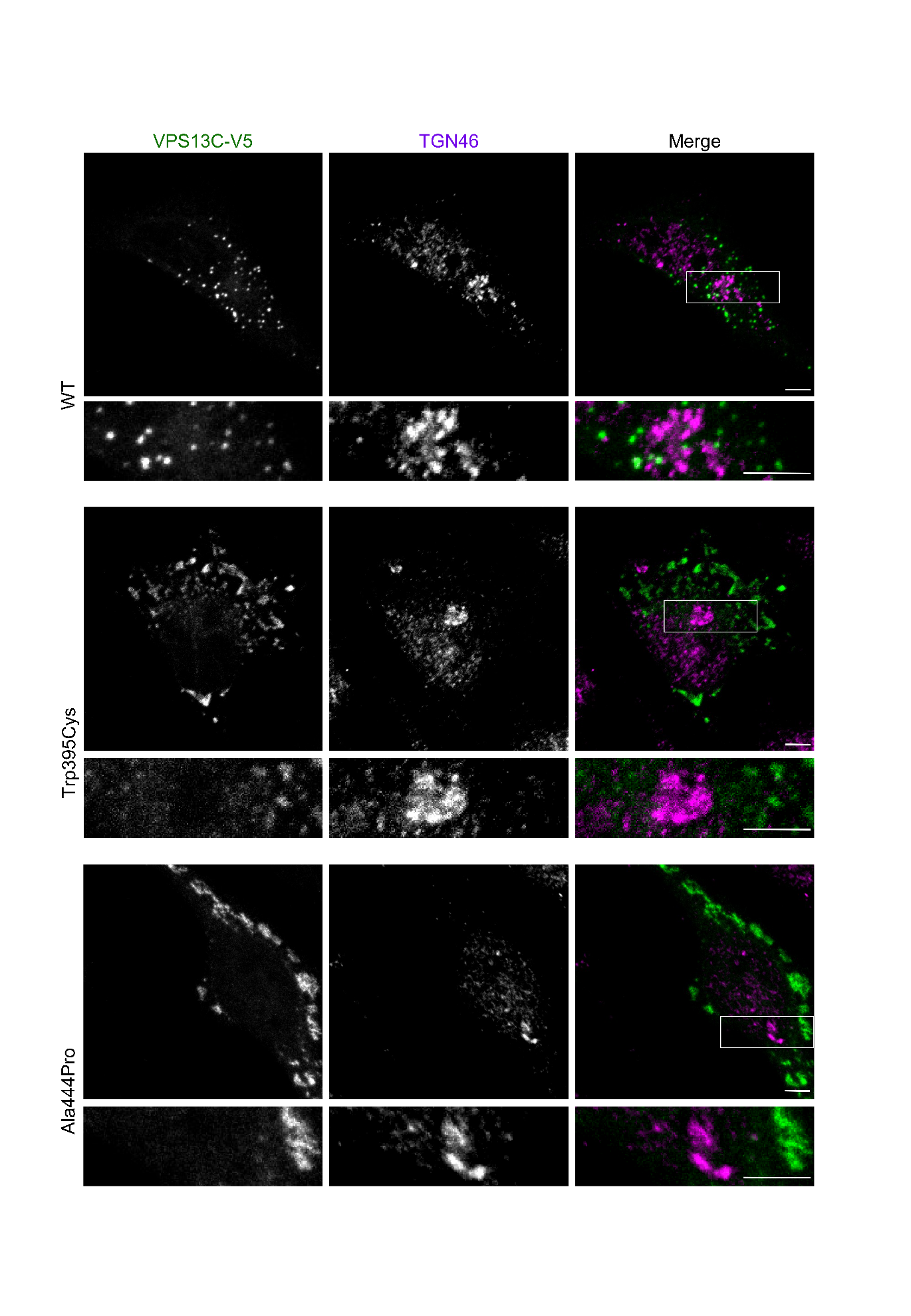


**Fig. S7. Wild type and p.Trp395Cys or p.Ala444Pro mutant VPS13C does not accumulate at the trans-Golgi.** HeLa cells were transfected with either wild type or mutant (p.Trp395Cys or p.Ala444Pro) VPS13C construct containing a C-terminal V5-tag. HeLa cells were double stained with an anti-V5-tag antibody to visualizeVPS13C (green) and an antibody staining the trans-Golgi (TGN46, magenta). No co-staining of VPS13C with the trans-Golgi could be observed for wild type or mutated VPS13C. White square = zoom. Scale bar = 5µm.

**Table S8. Primer sequences**

| **Allele specific PCR** | |
| --- | --- |
| **Name** | **Sequence** |
| VPS13C_I2789Twt_F | CAGCTGGATGTTTCACATGAA |
| VPS13C_I2789var_F | CAGCTGGATGTTTCACATGAG |
| VPS13C_I2789T_R | GCTGATGTTCTGCATTCGAG |
| VPS13C_M2711I_F | TTATGTGAACAAAACCACACCTT |
| VPS13C_M2711Iwt_R | CGAGAATCAGTGGTGAAATAATG |
| VPS13C_M2711Ivar_R | CGAGAATCAGTGGTGAAATAATA |
| **ONT long-read cDNA sequencing** | |
| VNP | 5phos/ACTTGCCTGTCGCTCTATCTTCTTTTTTTTTTTTTTTTTTTTVN |
| TSO | TTTCTGTTGGTGCTGATATTGCTGCCATTACGGCCmGmGmG |
| Forward primer | TTTCTGTTGGTGCTGATATTGC |
| Reverse primer | ACTTGCCTGTCGCTCTATCTTC |
| P5_ONT_F | ATCCAGATGCCACAGAAGGA |
| P5_ONT_R | TTCGGCAGTGTTTACATCCA |
| P6_ONT_F | CAGAGGTCCCAAATGAGGAG |
| P6_ONT_R | CAGGGAAGCCCATTACTCTG |
| P7_ONT_F | GGTTTCCACCATGAAAAGTCA |
| P7_ONT_R | ATCTCCATCCCTGGAATTTG |
| P12_ONT_F | CAGAGGTCCCAAATGAGGAG |
| P12_ONT_R | AGGGAACATTGCACCTGGTA |
| C2_ONT_F | TTGTTGTCCCTGGAGCAAGT |
| C2_ONT_R | GCCTGGACTGTGGCTGTACT |
| C7_ONT_F | GTGTTGACACGGCAACAGAA |
| C7_ONT_R | CCAGTACAACCTGGCCCTTA |
| **Generation of VPS13C antibody** | |
| **Name** | **Sequence** |
| VPS13C.2_F | GGAGCTAGCGAGAACCACATCAAGAAACTGG |
| VPS13C.2_R | CCGCTCGAGCTAAGACTGCTTCATCAGCTTTTGC |
| **Quantitative RT-PCR** | |
| **Name** | **Sequence** |
| VPS13C_qPCR_F | GAAAGAGTCTAAGAAAAAGGACGAAGA |
| VPS13C_qPCR_R | TCCTCTGGAGTCATAAGGTCATCA |
| **Splice site variant analysis on cDNA** | |
| **Name** | **Sequence** |
| VPS13C_c.4166-8C>A_F | CAAGGTGCCTGTTGTGGAAA |
| VPS13C_c.4166-8C>A_R | AGCTTCCATTCCAAGTTGCA |
| ***In vitro* mutagenesis** |  |
| **Name** | **Sequence** |
| VPS13C_Trp395Cys_F | CCCAGATGTGGTCCTGCTCCAACATCAAGAAGC |
| VPS13C_Trp395Cys_R | GCTTCTTGATGTTGGAGCAGGACCACATCTGGG |
| VPS13C_Ala444Pro_F | GTTCAACATCATCCTGCCCCGGCAGCAGGCCCAG |
| VPS13C_Ala444Pro_R | CTGGGCCTGCTGCCGGGGCAGGATGATGTTGAAC |

Abbreviations: ONT, Oxford Nanopore Technologies; VNP, VN primer: TSO, Template Switching Oligonucleotides.


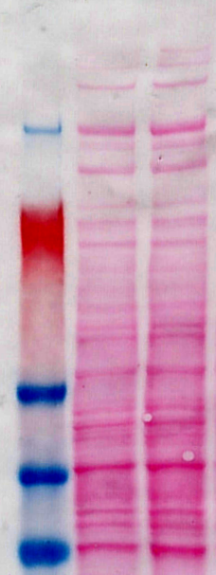

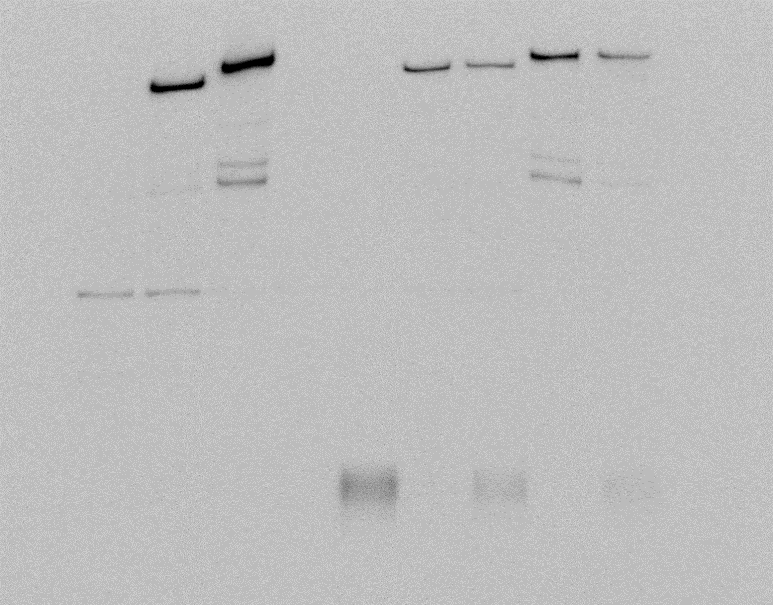


**HeLa**

**KO WT**

**HeLa**

**KO WT**

**kDa**

**210**

**111**

**71**

**55**

**41**

**Fig S8. Western blot analysis of extracts from VPS13C knockout and wild type HeLa cells using the affinity-purified VPS13C antibody.** Loaded extract: 10 µg. Ponceau S staining (right side) used as loading control.


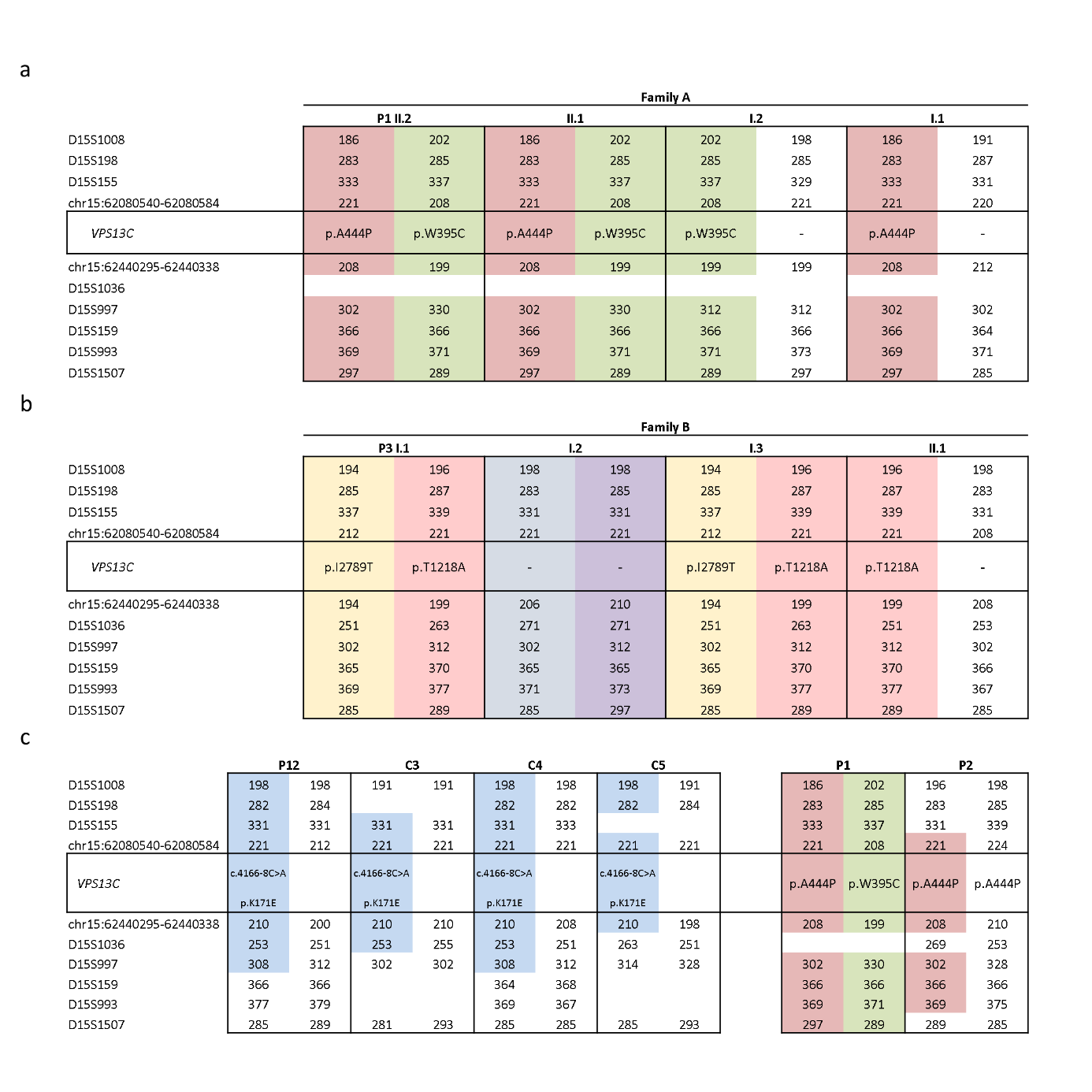


**Fig. S9. Haplotype sharing analysis.** Haplotype sharing analysis in family A (**a**) and family B (**b**) (Fig. 1) to confirm *trans* configuration of *VPS13C* mutant alleles. **c.** Patient and control carriers of *VPS13C* p.Lys171Glu/c.4166-8C>A share a common haplotype, colored in blue, indicating *cis* configuration of both variants. In patient P2, p.Ala444Pro is present on two different haplotypes, of which one haplotype shared with patient P1.


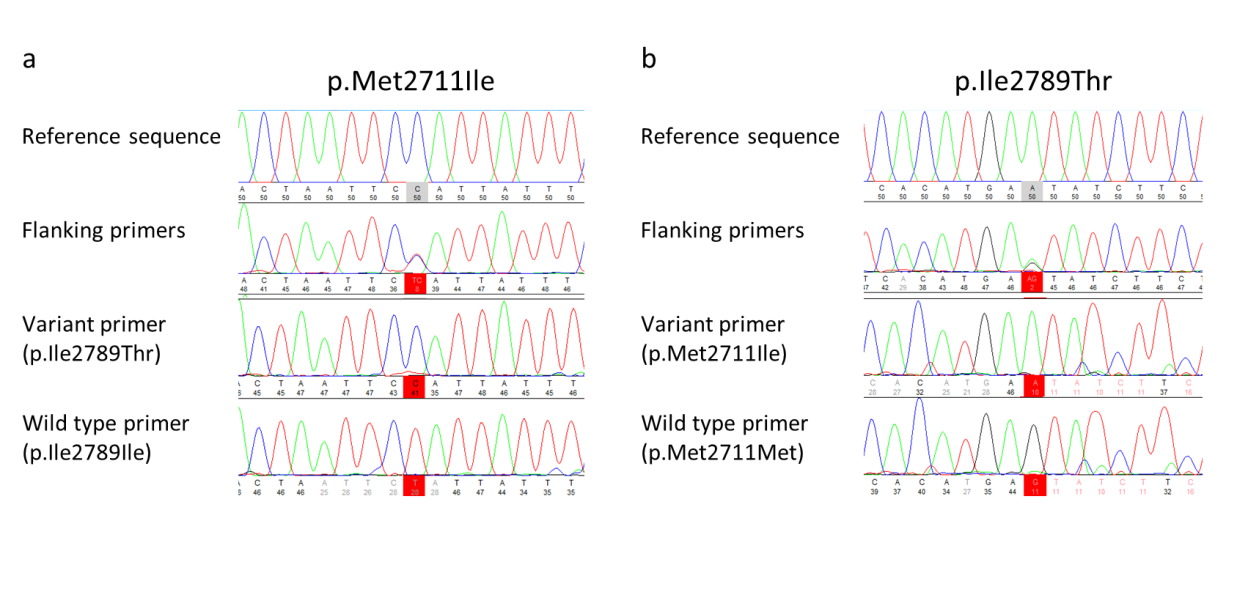


**Fig. S10. *Trans* configuration of p.Met2711Ile/p.Ile2789Thr in patient P4 confirmed by allele specific PCR. a.** Two different forward primers were designed: One containing the wild type nucleotide and one the variant nucleotide of p.Ile2789Thr. PCR amplification with the variant primer resulted in the absence of p.Met2711Ile, while amplification using the wild type primer resulted in an amplicon containing p.Met2711Ile. **b.** Likewise, two different reverse primers were designed: One containing the wild type nucleotide and one the variant nucleotide of p.Met2711Ile. PCR amplification with the variant primer resulted in the absence of p.Ile2789Thr and amplification using the wild type primer resulted in amplicons containing p.Ile2789Thr.


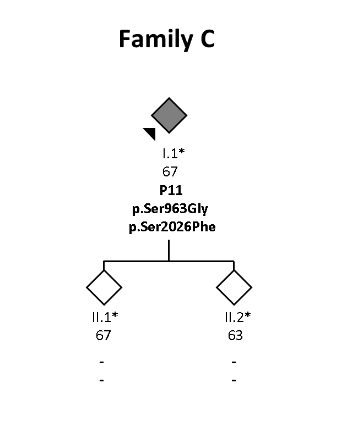


**Fig. S11. Family C.** One affected patient (P11) with compound heterozygous *VPS13C* missense mutations p.Ser963Gly and p.Ser2026Phe. Both children of patient P11 are negative for p.Ser963Gly and p.Ser2026Phe, suggesting *cis* configuration of the missense mutations in the patient. To make the pedigrees anonymous, we used diamonds for the family members and the patients (black symbol) and we added to the pedigree only family members needed to show the cis/*trans* location of the *VPS13C* missense mutations. Slashed symbols indicate deceased family members.

**
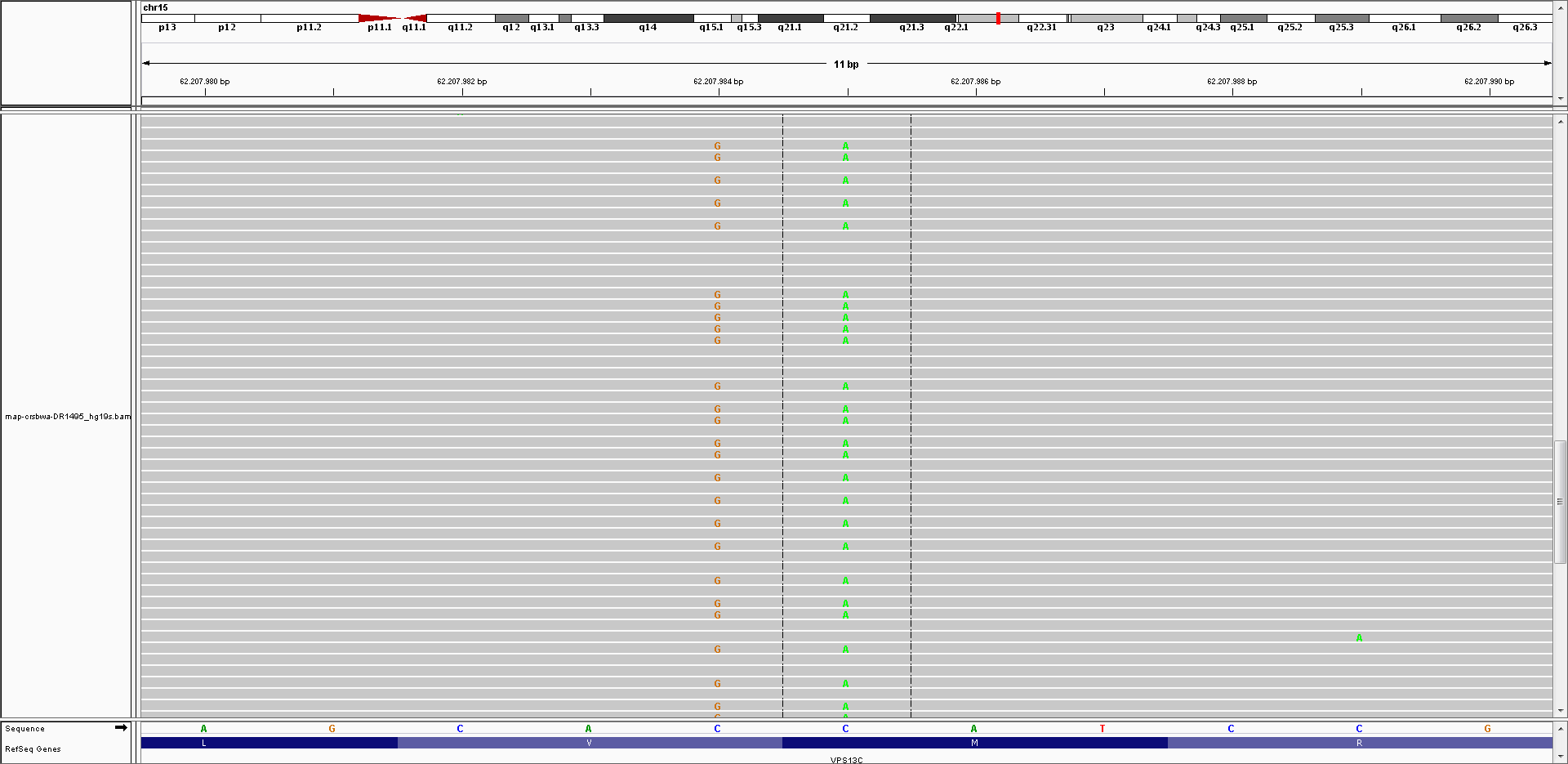
**

**Fig. S12. *Cis* configuration of p.Met2764Ile/p.Val2765Leu in control C6.** Sequencing reads generated via targeted resequencing of *VPS13C* were visualized with the Integrative Genomics Viewer (IGV) [21] using .bam files of individual samples. The heterozygous *VPS13C* p.Met2764Ile/p.Val2765Leu missense mutations in control carrier C6 are confirmed to be in *cis* configuration, by visualizing both missense mutations always together on sequencing reads.

**Table S9. *In-silico* predictions on *VPS13C* mRNA splicing of splice site variants in compound heterozygous carriers**

| **Splicing prediction method [Range], Threshold** | **c.448+7A>G** | | **c.4056+3A>C** | | **c.4166-8C>A** | |
| --- | --- | --- | --- | --- | --- | --- |
|  | **Reference** | **Mutated** | **Reference** | **Mutated** | **Reference** | **Mutated** |
|  | **c.448+7A** | **c.448+7G** | **c.4056+3A** | **c.4056+3C** | **c.4166-8C** | **c.4166-8A** |
| SSF [0-100], ≥ 70 | 77.05 | 77.05 (-0%) | 87.08 | 77.20 (-11.3%) | 85.25 | 81.88 (-4.0%) |
| MaxEnt [0-12], ≥ 0 | 6.29 | 6.29 (-0%) | 8.62 | 1.93 (-77.7%) | 9.00 | 8.64 (-4.0%) |
| NNSPLICE [0-1], ≥ 0.4 | 0.48 | 0 (-100%) | 0.99 | 0 (-100%) | 0.82 | 0 (-100%) |
| GeneSplicer [0-24], ≥ 0 |  |  |  |  | 2.83 | 1.57 (-44.6%) |

Note: Predicted effect on splicing due to *VPS13C* c.448+7A>G, c.4056+3A>C, c.4166-8C>A, according to four different splicing prediction tools integrated in the Alamut Visual version v.2.11.0 (Interactive Biosoftware, Rouen, France): SpliceSiteFinder-like (SSF), MaxEntScan (MaxEnt), NNSPLICE and GeneSplicer. Coding nomenclature is according to NM_025153. Splicing scores resulting from the reference and mutated sequences are listed within the complete range of possible values for each method. Only values passing the method-specific threshold are reported. The difference between the reference sequence score and the mutated sequence score are given in percentage.

**
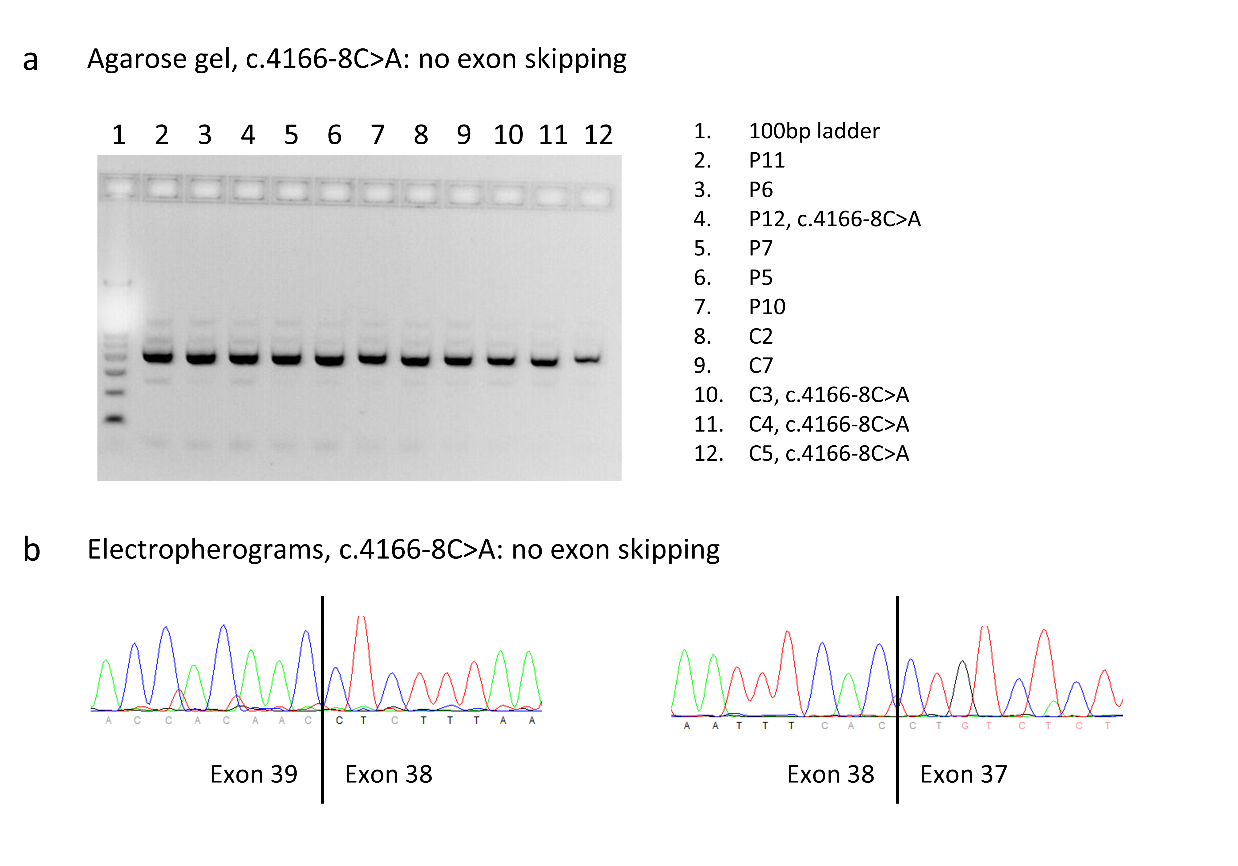
**

**Fig. S13. Effect of c.4166-8C>A on *VPS13C* mRNA splicing a.** Agarose gel electrophoresis of VPS13C cDNA amplicon with primers flanking exon 38. Exon skipping of exon 38 would result in a smaller amplicon of -123bp. No differences are observed between individuals with the c.4166-8C>A splice site variant and without. **b.** Sanger sequencing on cDNA of c.4166-8C>A carriers confirmed the presence of exon 38.

**LIST OF ABBREVIATIONS**

AAI, age at inclusion; AAO, age at onset; AD, Alzheimer’s disease; ALS, amyotrophic lateral sclerosis; CADD_Phred, Combined Annotation Dependent Depletion prediction score; CON, control; CSF, cerebrospinal fluid; CT, computerized tomography; DATscan, dopamine transporter imaging; DLB, dementia with Lewy bodies; Dx, diagnosis; EEG, electroencephalogram; F, frequency; FTD, frontotemporal dementia; GnomAD_nfe, Genome Aggregation Database non-Finnish European population; IGV, Integrative Genomics Viewer; L, left; MAF, minor allele frequency; MCI, mild cognitive impairment; MMSE, Mini-Mental State Examination; MRI, magnetic resonance imaging; NA, not available; ONT, Oxford Nanopore Technologies; PD, Parkinson’s disease; SPECT, single photon emission computed tomography; TSO, Template Switching Oligonucleotides; R, right; UPDRS, Unified Parkinson's Disease Rating Scale; VNP, VN primer; ∆CDS, coding sequence substitution; ΔAA, amino acid substitution.

**REFERENCES**

1. Auton A, Brooks LD, Durbin RM, Garrison EP, Kang HM, Korbel JO et al. (2015) A global reference for human genetic variation. Nature 526:68-74. doi:10.1038/nature15393

2. Brouwers N, Nuytemans K, van der Zee J, Gijselinck I, Engelborghs S, Theuns J et al. (2007) Alzheimer and Parkinson diagnoses in progranulin null mutation carriers in an extended founder family. Arch Neurol 64:1436-1446. doi:10.1001/archneur.64.10.1436

3. DePristo MA, Banks E, Poplin R, Garimella KV, Maguire JR, Hartl C et al. (2011) A framework for variation discovery and genotyping using next-generation DNA sequencing data. Nat Genet 43:491-498. doi:10.1038/ng.806

4. Drmanac R, Sparks AB, Callow MJ, Halpern AL, Burns NL, Kermani BG et al. (2010) Human genome sequencing using unchained base reads on self-assembling DNA nanoarrays. Science 327:78-81. doi:10.1126/science.1181498

5. Edge P, Bansal V (2019) Longshot enables accurate variant calling in diploid genomes from single-molecule long read sequencing. Nat Commun 10:4660. doi:10.1038/s41467-019-12493-y

6. Folstein MF, Folstein SE, McHugh PR (1975) "Mini-mental state". A practical method for grading the cognitive state of patients for the clinician. J Psychiatr Res 12:189-198. doi:10.1016/0022-3956(75)90026-6

7. Gijselinck I, van der Zee J, Engelborghs S, Goossens D, Peeters K, Mattheijssens M et al. (2008) Progranulin locus deletion in frontotemporal dementia. Hum Mutat 29:53-58. doi:10.1002/humu.20651

8. Goetz CG, Fahn S, Martinez-Martin P, Poewe W, Sampaio C, Stebbins GT et al. (2007) Movement Disorder Society-sponsored revision of the Unified Parkinson's Disease Rating Scale (MDS-UPDRS): Process, format, and clinimetric testing plan. Mov Disord 22:41-47. doi:10.1002/mds.21198

9. Hoehn MM, Yahr MD (1967) Parkinsonism: onset, progression and mortality. Neurology 17:427-442. doi:10.1212/wnl.17.5.427

10. Homig-Holzel C, Savola S (2012) Multiplex ligation-dependent probe amplification (MLPA) in tumor diagnostics and prognostics. Diagn Mol Pathol 21:189-206. doi:10.1097/PDM.0b013e3182595516

11. Kircher M, Witten DM, Jain P, O'Roak BJ, Cooper GM, Shendure J (2014) A general framework for estimating the relative pathogenicity of human genetic variants. Nat Genet 46:310-315. doi:10.1038/ng.2892

12. Kumar N, Leonzino M, Hancock-Cerutti W, Horenkamp FA, Li P, Lees JA et al. (2018) VPS13A and VPS13C are lipid transport proteins differentially localized at ER contact sites. J Cell Biol 217:3625-3639. doi:10.1083/jcb.201807019

13. Lange V, Bohme I, Hofmann J, Lang K, Sauter J, Schone B et al. (2014) Cost-efficient high-throughput HLA typing by MiSeq amplicon sequencing. BMC Genomics 15:63. doi:10.1186/1471-2164-15-63

14. Lek M, Karczewski KJ, Minikel EV, Samocha KE, Banks E, Fennell T et al. (2016) Analysis of protein-coding genetic variation in 60,706 humans. Nature 536:285-291. doi:10.1038/nature19057

15. Li H (2018) Minimap2: pairwise alignment for nucleotide sequences. Bioinformatics 34:3094-3100. doi:10.1093/bioinformatics/bty191

16. Li H, Durbin R (2010) Fast and accurate long-read alignment with Burrows-Wheeler transform. Bioinformatics 26:589-595. doi:10.1093/bioinformatics/btp698

17. Li H, Handsaker B, Wysoker A, Fennell T, Ruan J, Homer N et al. (2009) The Sequence Alignment/Map format and SAMtools. Bioinformatics 25:2078-2079. doi:10.1093/bioinformatics/btp352

18. McKenna A, Hanna M, Banks E, Sivachenko A, Cibulskis K, Kernytsky A et al. (2010) The Genome Analysis Toolkit: a MapReduce framework for analyzing next-generation DNA sequencing data. Genome Res 20:1297-1303. doi:10.1101/gr.107524.110

19. Nuytemans K, Meeus B, Crosiers D, Brouwers N, Goossens D, Engelborghs S et al. (2009) Relative contribution of simple mutations vs. copy number variations in five Parkinson disease genes in the Belgian population. Hum Mutat 30:1054-1061. doi:10.1002/humu.21007

20. Reumers J, De Rijk P, Zhao H, Liekens A, Smeets D, Cleary J et al. (2012) Optimized filtering reduces the error rate in detecting genomic variants by short-read sequencing. Nat Biotechnol 30:61-68. doi:10.1038/nbt.2053

21. Robinson JT, Thorvaldsdottir H, Wenger AM, Zehir A, Mesirov JP (2017) Variant Review with the Integrative Genomics Viewer. Cancer Res 77:e31-e34. doi:10.1158/0008-5472.CAN-17-0337

22. Rozen S, Skaletsky H (2000) Primer3 on the WWW for general users and for biologist programmers. Methods Mol Biol 132:365-386

23. Schindelin J, Arganda-Carreras I, Frise E, Kaynig V, Longair M, Pietzsch T et al. (2012) Fiji: an open-source platform for biological-image analysis. Nat Methods 9:676-682. doi:10.1038/nmeth.2019

24. Schneider VA, Graves-Lindsay T, Howe K, Bouk N, Chen HC, Kitts PA et al. (2017) Evaluation of GRCh38 and de novo haploid genome assemblies demonstrates the enduring quality of the reference assembly. Genome Res 27:849-864. doi:10.1101/gr.213611.116

25. Smit A, Hubley R, Green P (1996-2010) RepeatMasker Open-3.0.

26. Weckx S, Del-Favero J, Rademakers R, Claes L, Cruts M, De Jonghe P et al. (2005) novoSNP, a novel computational tool for sequence variation discovery. Genome Res 15:436-442. doi:10.1101/gr.2754005
